# Machine learning analysis of RB-TnSeq fitness data predicts functional gene modules in *Pseudomonas putida* KT2440

**DOI:** 10.1128/msystems.00942-23

**Published:** 2024-02-07

**Authors:** Andrew J. Borchert, Alissa C. Bleem, Hyun Gyu Lim, Kevin Rychel, Keven D. Dooley, Zoe A. Kellermyer, Tracy L. Hodges, Bernhard O. Palsson, Gregg T. Beckham

**Affiliations:** 1 Renewable Resources and Enabling Sciences Center, National Renewable Energy Laboratory, Golden, Colorado, USA; 2 Center for Bioenergy Innovation, Oak Ridge National Laboratory, Oak Ridge, Tennessee, USA; 3 Agile BioFoundry, Emeryville, California, USA; 4 Department of Bioengineering, University of California San Diego, La Jolla, California, USA; 5 Joint BioEnergy Institute, Emeryville, California, USA; 6 Department of Biological Engineering, Inha University, Incheon, Korea; 7 Novo Nordisk Foundation Center for Biosustainability, Technical University of Denmark, Lyngby, Denmark; 8 Department of Pediatrics, University of California, San Diego, California, USA; University of British Columbia, Vancouver, British Columbia, Canada

**Keywords:** transposon insertion sequencing, RB-TnSeq, independent component analysis, machine learning, *Pseudomonas putida*, aromatic catabolism, amino acid metabolism, functional genomics

## Abstract

There is growing interest in engineering *Pseudomonas putida* KT2440 as a microbial chassis for the conversion of renewable and waste-based feedstocks, and metabolic engineering of *P. putida* relies on the understanding of the functional relationships between genes. In this work, independent component analysis (ICA) was applied to a compendium of existing fitness data from randomly barcoded transposon insertion sequencing (RB-TnSeq) of *P. putida* KT2440 grown in 179 unique experimental conditions. ICA identified 84 independent groups of genes, which we call fModules (“functional modules”), where gene members displayed shared functional influence in a specific cellular process. This machine learning-based approach both successfully recapitulated previously characterized functional relationships and established hitherto unknown associations between genes. Selected gene members from fModules for hydroxycinnamate metabolism and stress resistance, acetyl coenzyme A assimilation, and nitrogen metabolism were validated with engineered mutants of *P. putida*. Additionally, functional gene clusters from ICA of RB-TnSeq data sets were compared with regulatory gene clusters from prior ICA of RNAseq data sets to draw connections between gene regulation and function. Because ICA profiles the functional role of several distinct gene networks simultaneously, it can reduce the time required to annotate gene function relative to manual curation of RB-TnSeq data sets.

**IMPORTANCE:**

This study demonstrates a rapid, automated approach for elucidating functional modules within complex genetic networks. While *Pseudomonas putida* randomly barcoded transposon insertion sequencing data were used as a proof of concept, this approach is applicable to any organism with existing functional genomics data sets and may serve as a useful tool for many valuable applications, such as guiding metabolic engineering efforts in other microbes or understanding functional relationships between virulence-associated genes in pathogenic microbes. Furthermore, this work demonstrates that comparison of data obtained from independent component analysis of transcriptomics and gene fitness datasets can elucidate regulatory-functional relationships between genes, which may have utility in a variety of applications, such as metabolic modeling, strain engineering, or identification of antimicrobial drug targets.

## INTRODUCTION

*Pseudomonas putida* KT2440 (hereafter, *P. putida*) has garnered much interest as a host for the valorization of heterogeneous chemical streams, such as biomass- and plastic-derived feedstocks, owing to its ability to adapt to several, often toxic, environments and funnel heterogeneous substrates toward a single product (1–9). However, a deep understanding of the metabolic and stress tolerance capabilities of *P. putida* is essential for its use as an industrial biocatalyst (10–15).

For all organisms, including *P. putida*, activities of multiple gene products must be coordinated to form complex functional networks that permit survival and growth within the environments experienced by the cell, and metabolic engineering efforts do not always account for the complex, inter-related nature of these gene networks (16). One approach to illuminate these relationships is transposon insertion sequencing (TnSeq), a powerful functional genomics tool that combines high-density transposon mutagenesis with next-generation sequencing to simultaneously characterize bacterial gene essentiality and fitness (17, 18). By screening a pooled transposon library against various growth conditions, a TnSeq practitioner can establish the relative essentiality of each gene across the conditions tested, thereby generating a genotype-phenotype link that can help to elucidate the function of each feature. A variation on the TnSeq method, randomly barcoded transposon insertion sequencing (RB-TnSeq), uses unique barcode sequences encoded within transposons to reduce the sequencing burden of traditional TnSeq approaches (19). To date, RB-TnSeq has aided in the elucidation of the catabolism of aromatic acids, alcohols, fatty acids, lysine, and various nitrogen sources in addition to probing gene involvement during ionic, aromatic, and aliphatic acid stress in *P. putida* (20–24).

Nonetheless, while RB-TnSeq can aid in the assignment of gene function, this technique is restricted to the range of conditions studied, and identification of functionally related gene groups is often assessed through manual curation of the data sets. In contrast to principle component analysis (PCA), which uses dimensionality reduction to compress multivariate information, independent component analysis (ICA) is an unsupervised, multivariate signal separation algorithm used to decompose mixed signals into their individual parts (25). This approach performs best among many algorithms to identify sets of co-expressed genes (26) and has been successfully applied to microarray and RNAseq transcriptomics data (27–32).

In this work, ICA was applied to gene fitness data obtained from a set of diverse, previously conducted RB-TnSeq experiments, enabling rapid deconvolution of the complex genetic network of *P. putida* into groups of functionally independent genes. These functional groups, named fModules (for “functional modules”), represent sets of co-functioning genes that show correlated fitness performance across all conditions in the RB-TnSeq data set. The function of several genes was then examined *in vivo* to validate fModule membership. These included genes involved in hydroxycinnamate metabolism and tolerance, nitrogen assimilation from amino acid substrates, and acetyl-coenzyme A (CoA) utilization. The functional clustering data obtained from ICA of RB-TnSeq data sets were also compared with prior gene regulatory clustering data to establish regulation-function relationships between sets of genes in *P. putida* (32).

## RESULTS AND DISCUSSION

### ICA of multivariate gene fitness data separates genes into fModules

The appropriateness of applying ICA to gene fitness data was assessed through review of the required assumptions for the approach. ICA assumes that (i) independent components are statistically independent and (ii) independent components have a non-Gaussian distribution. For the first criterion, the statistical independence of functional gene networks seems counterintuitive given that the metabolic network consists of sets of intersecting anabolic, catabolic, and energy transfer reactions (33). However, empirical results have shown that ICA estimation of meaningful components is robust against some violation of the independence assumption (34). In practice, this means that ICA allows for variable membership to multiple independent components (as with metabolic intersection) but requires that interactions among members of an independent component be stronger than interactions between components to be effective. Therefore, the modularity of functional processes, like those within the metabolic network satisfies the requirement for independence (33, 35). This is similar to the successful application of ICA to expression data based upon the modularity of regulatory networks, where large sets of genes are transcriptionally controlled by relatively independent sets of global regulators that, in some cases, can also control the expression of other global regulators (regulatory intersection) (30).

The second requirement for non-Gaussian distribution of the independent components is also satisfied by the modularity of physiological processes, where the fitness distribution of a mutant strain is driven to non-Gaussian behavior, based upon the functional role of the gene product in each growth condition. For example, if a gene plays a functional role in efflux of a toxic compound, fitness outcomes from disrupting that gene would be skewed negative during growth in the presence of the toxic compound but neutral in conditions where the toxin is absent. If several genes are required for growth in the presence of the toxin, then they will exhibit similar, non-Gaussian distributions in the data set and be grouped together into a single fModule.

**Fig 1:**
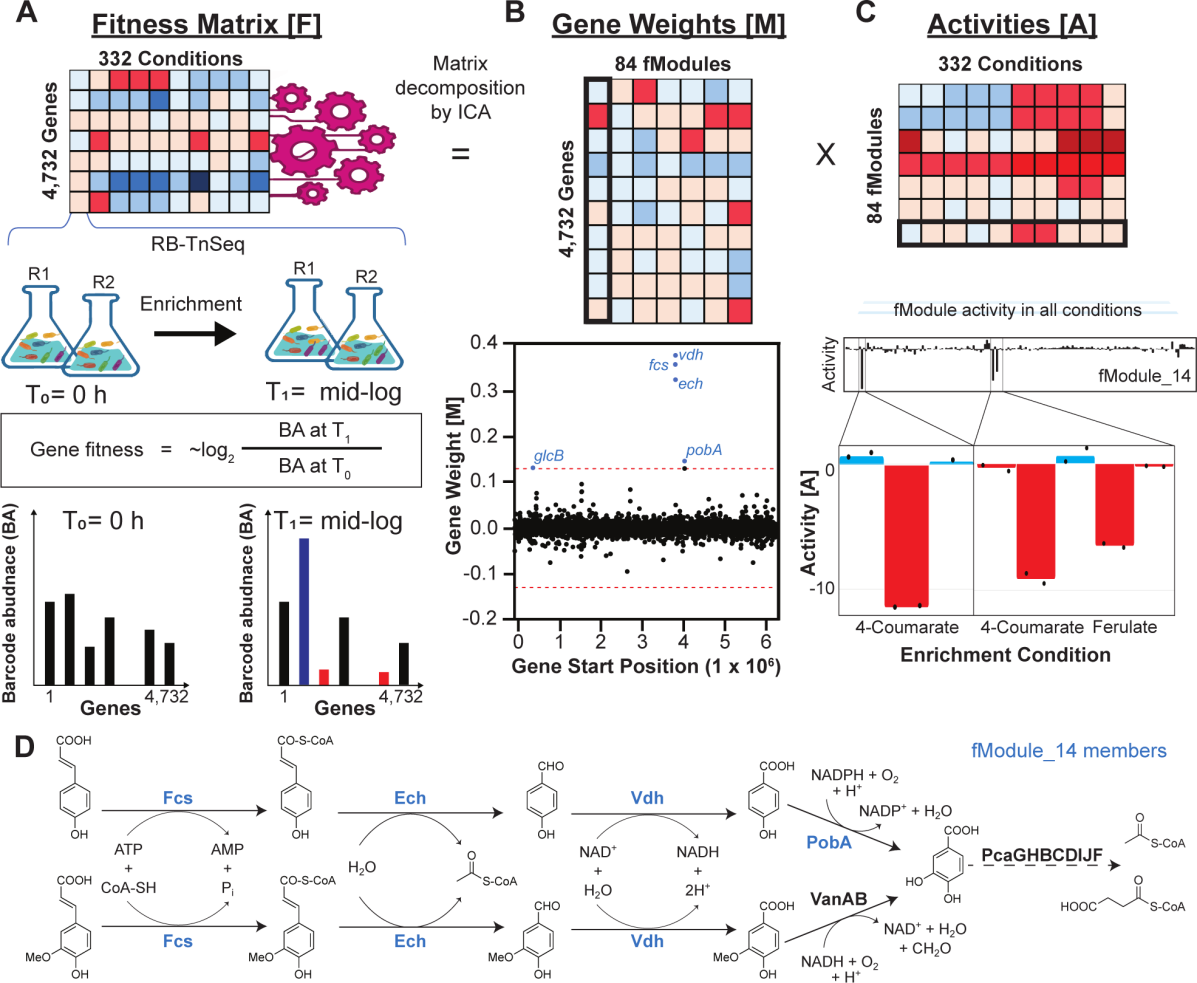
Decomposition of RB-TnSeq multivariate gene fitness profiles using ICA. (**A**) Schematic of the general RB-TnSeq approach. A randomly barcoded transposon insertion library in *P. putida* is cultivated in a baseline (“time zero”, T_0_) condition and then enriched in a selective condition to mid-log (T_1_). Normalized barcode counts, which are indicative of specific mutant frequency in the population, are enumerated before and after enrichment to calculate a fitness value for each gene disruption. Genes with larger fitness magnitudes are expected to play a more important functional role for a given condition. In ICA, (**A**) fitness values, $\left[F\right]$, from RB-TnSeq experiments were decomposed into a matrix of (**B**) gene weights, $\left[M\right]$, and a matrix of (**C**) condition-specific activities, $\left[A\right]$ . In $\left[M\right]$, genes with outlier weight values were grouped together into a single functional group, termed a “functional module”, which we abbreviate as “fModule”. In $\left[A\right]$, the activity profile of an fModule characterizes the related biological functions of its member genes in each RB-TnSeq enrichment condition. Data from fModule_14 is provided as an example of ICA fModule gene weight and fModule activity outputs. (**D**) fModule_14 contains several genes involved in hydroxycinnamate metabolism. The negative activity of fModule_14 for conditions with 4*-*coumarate or ferulate as a sole carbon source in panel **C** is consistent with the essential role of its member gene products in catabolism of these substrates.

With the applicability of ICA of gene fitness data established, a broad panel of publicly available RB-TnSeq data from *P. putida* was compiled into a set of gene fitness measurements, covering 4,732 of 5,564 protein-coding genes in *P. putida* (36) from 332 samples grown in 179 unique conditions (Fig. 1A; see “Data Availability”). Gene fitness values were derived from a variety of selection conditions, including growth on single carbon sources, growth on single nitrogen sources, metabolite and osmotic stress, and volumetric scales ranging from microtiter plates to 2-L bioreactors (20–23, 37, 38). This data set was then subjected to matrix decomposition by ICA to obtain individual groups of genes unified by shared functional influence upon specific cellular processes.

When ICA was applied to the *P. putida* RB-TnSeq data set, $\left[F\right]$, gene fitness data were decomposed into a matrix of individual gene weight coefficients, [M], for 84 underlying fitness profiles (fModules) (Fig. 1B), and a matrix of condition-specific activities, $\left[A\right]$, for each fModule (Fig. 1C). Most gene weight coefficients in an fModule were near zero, indicating that the underlying functional signal corresponding to each fModule affected a small number of significant genes. Genes with weight coefficients outside a predetermined threshold (see “Materials and Methods”) were removed, resulting in a set of outlier genes, which were identified as “member genes” for each fModule. Member genes with negative or positive gene weights displayed fitness profiles negatively or positively correlated with fModule activity, respectively. For example, if an fModule displayed negative activity for a particular condition, transposon-mediated disruption of its positively weighted member genes would be detrimental to growth in that condition. In this way, underlying fitness values were decomposed into a matrix of activities, $\left[A\right]$, which reflected the concerted changes in fitness displayed by all member genes for each condition. In this analysis, only fModule_80 failed to contain a gene that satisfied the predetermined weight cutoff, resulting in an fModule with zero members. The 84 fModules contained 543 of the 4,732 unique genes used as input [11% of input, 9% of all *P. putida* open reading frames (ORFs)] with a median of 7 genes per fModule. In total, 83% of gene fitness variance within the RB-TnSeq data set was explained by the 84 fModules, and the proportion of total RB-TnSeq data set fitness variance explained by each fModule ranged from 0.14% to 8.47% (Table 1).

**TABLE 1:** fModule annotation and explained variance^*a*^

*fModule*	Description	# of genes	Explained variance	fModule	Description	# of genes	Explained variance
1	Fatty Acid Metabolism	3	0.21%	43	Phenylalanine Catabolism	11	0.18%
2	Nitrogen metabolism	6	2.63%	44	Beta-alanine catabolism	10	0.31%
3	RidA	1	0.98%	45	Spermidine and propandiamine catabolism	17	0.27%
4	Transcriptional regulation	13	0.32%	46	Butyrolactam catabolism	5	0.15%
5	Benzoate catabolism	13	0.23%	47	Transcriptional regulation and cell:surface adhesion	6	0.65%
6	Glycine, serine, and threonine metabolism	9	0.14%	48	Acetic acid stress	7	0.57%
7	Choline, betaine, and carnitine catabolism	22	0.22%	49	4-Hydroxyvalerate catabolism	2	0.45%
8	Propanediamine catabolism	22	0.61%	50	1,4-Butanediol catabolism	7	0.34%
9	Maintenance of lipid asymmetry (Mla) system	14	0.50%	51	Phenolic acid stress	19	0.24%
10	Bioreactor growth	3	0.40%	52	Flagellar biosynthesis	25	0.39%
11	Levulinic acid catabolism	16	0.52%	53	Fatty acid metabolism	11	0.88%
12	Biotin biosynthesis and pyruvate carboxylase	9	1.24%	54	ParB	1	0.29%
13	Molybdopterin biosynthesis and benzaldehyde tolerance	20	0.42%	55	Lactic acid stress	14	0.78%
14	4-Coumarate and ferulate catabolism	5	0.15%	56	Thymine degradation	6	0.20%
15	Glutamate metabolism	3	1.09%	57	Branched-chain Amino acid biosynthesis	5	7.48%
16	Benzoate, 4-coumarate, and ferulate catabolism	16	1.96%	58	Fatty acid catabolism	4	0.64%
17	Pyrroloquinoline biosynthesis and short-chain alcohol catabolism	20	0.41%	59	Thiamine diphosphate biosynthesis	6	2.45%
18	Phenylacetate catabolism	16	0.30%	60	4-Aminobutyric acid catabolism	7	0.18%
19	Phosphoenolpyruvate:sugar phosphotransferase system	2	0.37%	61	Nitrogen metabolism	6	0.20%
20	4-Coumarate, ferulate, and catabolic intermediate stress tolerance	6	0.34%	62	Galacturonic acid and glucuronic acid catabolism	11	0.26%
21	Arginine metabolism	14	3.12%	63	Methionine biosynthesis	5	3.80%
22	Cobalamin biosynthesis and ethanolamine catabolism	22	0.50%	64	NadC	1	2.51%
23	Uncharacterized	4	0.71%	65	Glutamate transport	12	0.36%
24	Nitrogen stress sensor and glycogen biosynthesis	3	0.43%	66	Proline metabolism	2	2.05%
25	Bioreactor growth	14	0.68%	67	Valine biosynthesis	3	3.71%
26	Uncharacterized	11	0.27%	68	Lysine metabolism	18	0.60%
27	Membrane to surface adhesion	5	0.96%	69	Histidine biosynthesis	2	3.82%
28	Ferulate and catabolic intermediate catabolism	9	0.32%	70	Butyrate catabolism	9	0.34%
29	Transport of butylamine-containing compounds	6	0.16%	71	HisA	1	0.50%
30	Histidine biosynthesis	11	6.89%	72	Lysine metabolism	5	0.27%
31	Protocatechuate stress	8	0.58%	73	L-Lysine catabolism	3	0.24%
32	Nitrogen assimilation	4	0.95%	74	Tryptophan biosynthesis	9	8.47%
33	Biotin biosynthesis and isopentanol/isoprenol catabolism	12	0.35%	75	Uncharacterized	6	1.86%
34	Improved fitness factors	7	1.91%	76	Sodium tolerance	21	0.58%
35	Uncharacterized	6	1.12%	77	Fatty acid metabolism	2	0.15%
36	Possible Tween-20 catabolism	12	0.24%	78	Possible butyrate metabolism	7	0.18%
37	Lipopolysaccharide biosynthesis	12	0.27%	79	Fatty ester metabolism	7	0.32%
38	RuvC	1	0.37%	80	No gene	0	0.21%
39	PP_1410	1	0.18%	81	SerA	1	1.32%
40	Acetate catabolism	3	0.23%	82	ProI	1	0.44%
41	Hexanoate and valerate catabolism	10	0.20%	83	Beta-ketoadipate and levulinic acid stress	4	0.22%
42	NADH:ubiquinone oxidoreductase I	14	1.68%	84	Nitrate metabolism	3	0.15%

^*a*^ Functional annotations for each fModule, the number of genes in each fModule, and the proportion of the RB-TnSeq data set variance explained by each fModule. 543 unique genes are contained within the 84 fModules. Full details for each fModule are available at https://fmodules.github.io/putida.

The functional role of each fModule was initially classified using the Database for Annotation, Visualization, and Integrated Discovery (DAVID) database functional annotation clustering tool (39–41). The fModules were then manually curated using Kyoto Encyclopedia of Genes and Genomes (KEGG) orthology designations and fModule activity patterns to assign a putative functional annotation for all fModules (42). An example of this process is provided for fModule_14, which contained five genes (Fig. 1B). DAVID was unable to assign a functional clustering annotation, but fModule_14 activity was negative for growth enrichments containing 4*-*coumarate or ferulate as a sole carbon source (Fig. 1C), and GO terms revealed that 4/5 genes played known roles in hydroxycinnamate catabolism (Fig. 1D). These findings led to fModule_14 being provided with the functional annotation of “4*-*coumarate and ferulate catabolism” (File S1). Table 1 provides a list of all 84 fModules with putative functional annotations and the explained variance for each fModule. Most fModules could be assigned a functional annotation, but four fModules were left uncharacterized, due to ambiguity associated with member genes and corresponding fitness profiles (Table 1). Overall, over 57% of variance was explained by fModules annotated as amino acid metabolism, carbon metabolism, and nitrogen metabolism (Fig. S2). This is consistent with the fact that most conditions in the RB-TnSeq data set were designed to screen against various sole nitrogen sources, carbon sources, or amino acid dropout media compositions. Full details for each fModule, including gene weights, $\left[M\right]$, and fModule activities, $\left[A\right]$, are available at https://fmodules.github.io/putida.

Previous work applying ICA to *E. coli* transcriptomics data sets revealed that increasing the number and diversity of samples analyzed by ICA leads to the identification of a greater number of gene modules, and these modules are of higher quality (31). Since the application of ICA to gene fitness data sets does not mathematically differ from its application to transcriptomics data sets, the number and quality of identified fModules characterized are also expected to increase as sample size of the input data set increases. Therefore, future studies appending additional conditions to the data set may further refine *P. putida* fModule abundance and quality described in this work.

Also of note, the mariner transposon used in the KT2440 library does not contain an outward-facing promoter, making polar mutations even more likely in situations where a disrupted gene is encoded within an operon. This is a potential pitfall of transposon mutagenesis, in which affected genes may display strong fitness scores despite a lack of true involvement in a biological process. fModules may therefore include a small number of physiologically irrelevant genes, underscoring the need for careful consideration of gene context in any reverse-engineering campaign based on ICA assignments.

### ICA successfully identifies several well-established metabolic pathways as fModules

Many fModules contained genes with well-characterized shared functional roles, underscoring the utility of ICA for grouping genes according to common function. For example, fModule_21 contained genes with described roles in L-arginine biosynthesis, transport, catabolism, and regulation (File S1) (43). The arginine biosynthesis genes displayed positive gene weights, while all other genes displayed negative gene weights. Accordingly, fModule_21 exhibited negative activity in all conditions lacking L-arginine supplementation (Fig. S3). In another example, fModule_68 and fModule_73, which exhibited negative activity when L-lysine, D-lysine (D-Lys), or catabolic intermediates were used as carbon or nitrogen sources (Fig. S4), contained genes with recently described roles in lysine regulation, transport, and catabolism (File S1) (20). In an example unrelated to amino acid metabolism, fModule_5 contained genes with characterized roles in benzoate catabolism (File S1) and displayed negative activity in conditions where benzoate was a sole carbon source (Fig. S5). Interestingly, negative weight gene members of fModule_5 were involved in the catabolism of protocatechuate toward the same product from benzoate catabolism, β-ketoadipate enol-lactone (44).

An additional, notable example of ICA successfully grouping enzymatically distinct but functionally related genes was demonstrated by fModule_28 (File S1). fModule_28 exhibited strongly negative activity for growth on the *O*-methylated aromatic compounds ferulate, vanillate, and vanillin (Fig. S6). Accordingly, this fModule contained *vanAB*, encoding the vanillate *O-*demethylase essential for growth in these conditions (45, 46). Formaldehyde is liberated as a product of *O-*demethylation by the native Rieske non-heme iron monooxygenase system, VanAB (46–48), and fModule_28 also includes *frmA* (encoding a glutathione-dependent formaldehyde dehydrogenase). Consistent with the glutathione and zinc cofactor requirements of FrmA, *gshB* (PP_4993, glutathione synthetase) and *znuB1* (PP_0117, inner membrane pore of the zinc ABC transporter (49)) were also members of fModule_28. Disruption of *znuB1* presented complexity due to its in-frame arrangement with *znuC1* (PP_0118), but disruption of the transporter binding protein, *znuA1* (PP_0120), led to poor growth with vanillate, relative to the wild-type, in the presence of zinc (Fig. S7).

Overall, the examples from arginine, lysine, benzoate, and catabolism of *O*-methylated aromatic substrates above demonstrate that ICA applied to fitness data can identify sets of genes with known functional relationships. Additionally, while the gene annotations of member genes within fModule_21, fModule_68 and fModule_73 together, fModule_5, and fModule_28 could be used to assign functional roles for these fModules in arginine metabolism, lysine catabolism, benzoate catabolism, and *O*-methylated aromatic catabolism, respectively, the activity profiles for these fModules were consistent with their functional annotations (Fig. S3 to S6), underscoring the power in using fModule activity profiles to assign function.

### ICA predicts genes with complementary roles in otherwise well-characterized functional groups

**Fig 2:**
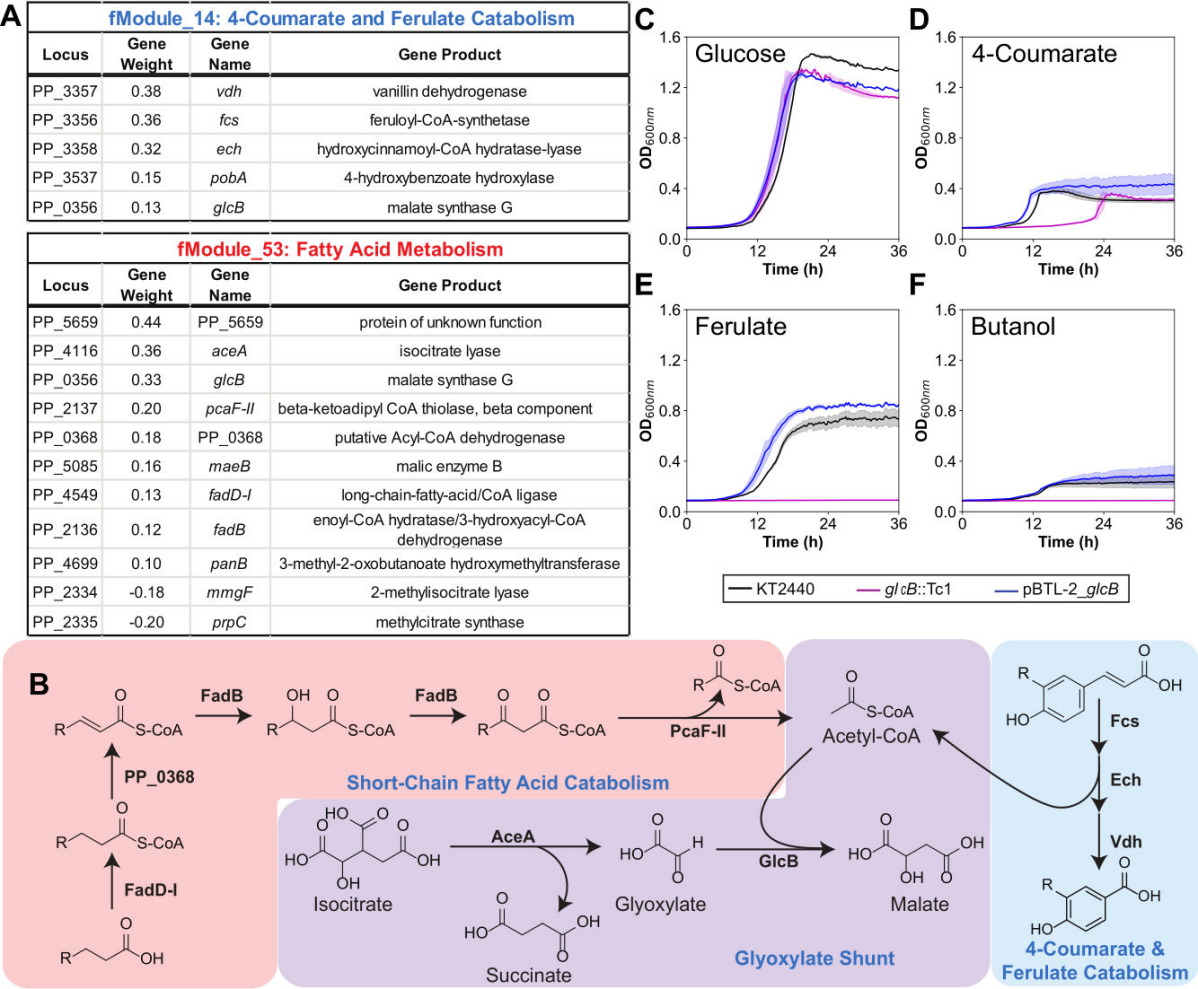
GlcB plays an important functional role within short-chain fatty acid metabolism and 4-coumarate and ferulate catabolism. (**A**) Membership and associated gene weights for fModule_14 and fModule_53. (**B**) Metabolic pathways for *P. putida* short-chain fatty acid metabolism, the glyoxylate shunt, and 4-coumarate (R=H) and ferulate (R=OCH_3_) catabolism. Growth profiles of a functional knockout of *glcB* (*glcB*::Tc1) and an overexpression mutant (pBTL-2_*glcB*) were compared with wild-type (KT2440) in M9 minimal medium supplemented with (**C**) 20 mM glucose, (**D**) 10 mM 4-coumarate, (**E**) 10 mM ferulate, and (**F**) 10 mM butanol. Error shading indicates the standard deviation from the mean for three biological replicates.

In several cases, the function of an fModule could be assigned based upon established roles for its constituent genes in a well-characterized pathway, but it also contained one or more genes with unintuitive roles in the pathway. One example of this observation is the inclusion of *glcB* (PP_0356), annotated as a malate synthase, in the well-characterized functional gene groups for fatty acid metabolism (fModule_53) and 4-coumarate and ferulate catabolism (fModule_14) (Fig. 2A; File S1). fModule_53 also included *aceA*, which together with *glcB* forms the glyoxylate shunt pathway to assimilate acetyl-CoA into the tricarboxylic acid (TCA) cycle (50). In conditions where catabolism of substrates results in the production of acetyl-CoA, such as with butanol and acetate, organisms are required to divert flux away from the oxidative steps of the TCA cycle and toward the anaplerotic steps of the glyoxylate shunt, conserving carbon for gluconeogenesis and subsequent biomass production (51). Accordingly, the abundance of glyoxylate shunt enzymes has been shown to increase in response to butanol (52), helping to explain the presence of *glcB* and *aceA* within fModule_53 for fatty acid metabolism.

Upregulation of the glyoxylate shunt genes has also been demonstrated during benzoate degradation, likely as a means to assimilate the acetyl-CoA generated by PcaF (53). Acetyl-CoA is also a product of the Fcs:Ech:Vdh pathway for 4-coumarate and ferulate catabolism (Fig. 2B), and the genes for these enzymes are members of fModule_14, along with *glcB* (Fig. 2A). The membership of *glcB* in both fModule_53 and fModule_14 was bolstered by growth experiments, where the transposon disruption mutant of *glcB* grew no different from wild-type *P. putida* in minimal medium supplemented with glucose (Fig. 2C), but its growth was severely inhibited on 4-coumarate (Fig. 2D) and ferulate (Fig. 2E; Fig. S8) and entirely abolished on butanol (Fig. 2F). Expression of additional copies of *glcB* from a plasmid (strain ACB329; Table S1) led to modest growth improvements on 4*-*coumarate and ferulate but did not affect growth on glucose or butanol, relative to the wild-type (Fig. 2C through F, blue lines). The poor growth of the functional *glcB* knockout strain on 4-coumarate and ferulate indicates a critical role for the glyoxylate shunt in facilitating the anaplerotic assimilation of acetyl-CoA generated during 4-coumarate and ferulate catabolism. Importantly, since catabolism of 4-coumarate or ferulate produces both acetyl-CoA and succinyl-CoA products (53), the glyoxylate shunt is not essential to the same degree as with the catabolism of butanol and acetate, where acetyl-CoA is the sole product. Nonetheless, engineering strategies that leverage *glcB* may offer an underutilized, broadly applicable approach to improve growth outcomes for processes that rely upon the catabolism of feedstocks containing ferulate or 4-coumarate.

### ICA aids in reannotation of gene function

**Fig 3:**
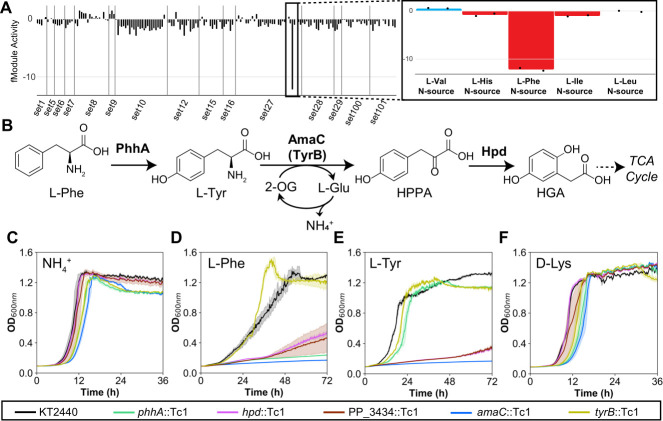
ICA suggests reannotation of AmaC from a D-lysine aminotransferase to an L-tyrosine aminotransferase. (**A**) Activity profile of fModule_43 across all conditions in the RB-TnSeq data set, with the inset showing strongly negative activity during growth with L-Phe as a nitrogen source. (**B**) Proposed catabolic pathway for L-Phe and L-Tyr in *P. putida*. The growth of individual transposon disruption mutants was compared with wild-type (KT2440), using M9 minimal medium supplemented with 20 mM glucose as a carbon source and either (**C**) 5 mM ammonium, (**D**) 5 mM L-Phe, (**E**) 5 mM L-Tyr, or (**F**) 5 mM D-Lys as the sole nitrogen source. Error shading indicates the standard deviation from the mean of three biological replicates. 2-OG, 2-oxoglutarate; HPPA, 4-hydroxyphenylpyruvic acid; HGA, homogentisic acid.

Occasionally, gene annotation seemed to be in conflict with a functional annotation derived from ICA. The membership of *amaC* (PP_3590, annotated as a D-lysine aminotransferase), in the phenylalanine catabolism functional module (fModule_43) serves as one example. fModule_43 exhibited a strongly negative activity during growth with L-phenylalanine (L-Phe) as the nitrogen source, so it was putatively annotated as a functional gene module for phenylalanine catabolism (Fig. 3A; Table 1). L-Phe catabolism proceeds through L-tyrosine (L-Tyr) in *P. putida* (Fig. 3B), but L-Tyr was not examined as a nitrogen source in the RB-TnSeq data set. To validate the role of genes included in fModule_43 (file S1) in L-Phe catabolism, single transposon disruption mutants of member genes *phhA*, *amaC*, and *hpd* were cultivated in M9 minimal medium with 20 mM glucose as a carbon source and either ammonium, L-glutamate (L-Glu), L-Phe, L-Tyr, or D-Lys as the sole nitrogen source (Fig. 3C through F). As expected, all mutants grew similarly to the wild-type strain when ammonium was used as the nitrogen source (Fig. 3C), and only a slight increase in lag time was observed for all mutants, except *hpd* when L-glutamate, a product of L-tyrosine aminotransferase, was used as the nitrogen source (Fig. S9). A substantial growth defect was observed when functional knockouts of *phhA*, *hpd*, and PP_3434 were grown with L-Phe as the sole source of nitrogen (Fig. 3D). The PP_3434 gene lies immediately upstream of *hpd* and presumably elicits a polar effect on the expression of *hpd*, but this was not explored further in the current work. While Hpd acts downstream of nitrogen assimilation within the L-Phe and L-Tyr catabolic pathway, it is possible that 4-hydroxyphenylpyruvic acid accumulation inhibits the activity of the upstream L-Tyr aminotransferase. This is bolstered by prior work, where an *hpd* knockout was used to increase the L-tyrosine concentration in *P. putida* (54). In accordance with the function of PhhA as a phenylalanine-4-hydroxylase, disruption of *phhA* did not substantially inhibit growth on L-Tyr (Fig. 3E). Surprisingly, disruption of *tyrB* (PP_1972, annotated as an L-Tyr aminotransferase) did not inhibit growth with L-Phe or L-Tyr, but disruption of *amaC* [PP_3590, sometimes called *tyrB2* (55)] completely abrogated growth with L-Phe and L-Tyr (Fig. 3D and E) and did not substantially inhibit growth on D-Lys (Fig. 3F), inconsistent with its annotation as a D-lysine aminotransferase. Given these results and findings from previous work suggesting that AmaD, not AmaC, is the predominant D-lysine aminotransferase in *P. putida* (20, 55), we propose re-annotation of AmaC as an L-Tyr aminotransferase.

### fModules uncover previously uncharacterized functional relationships between genes

**Fig 4:**
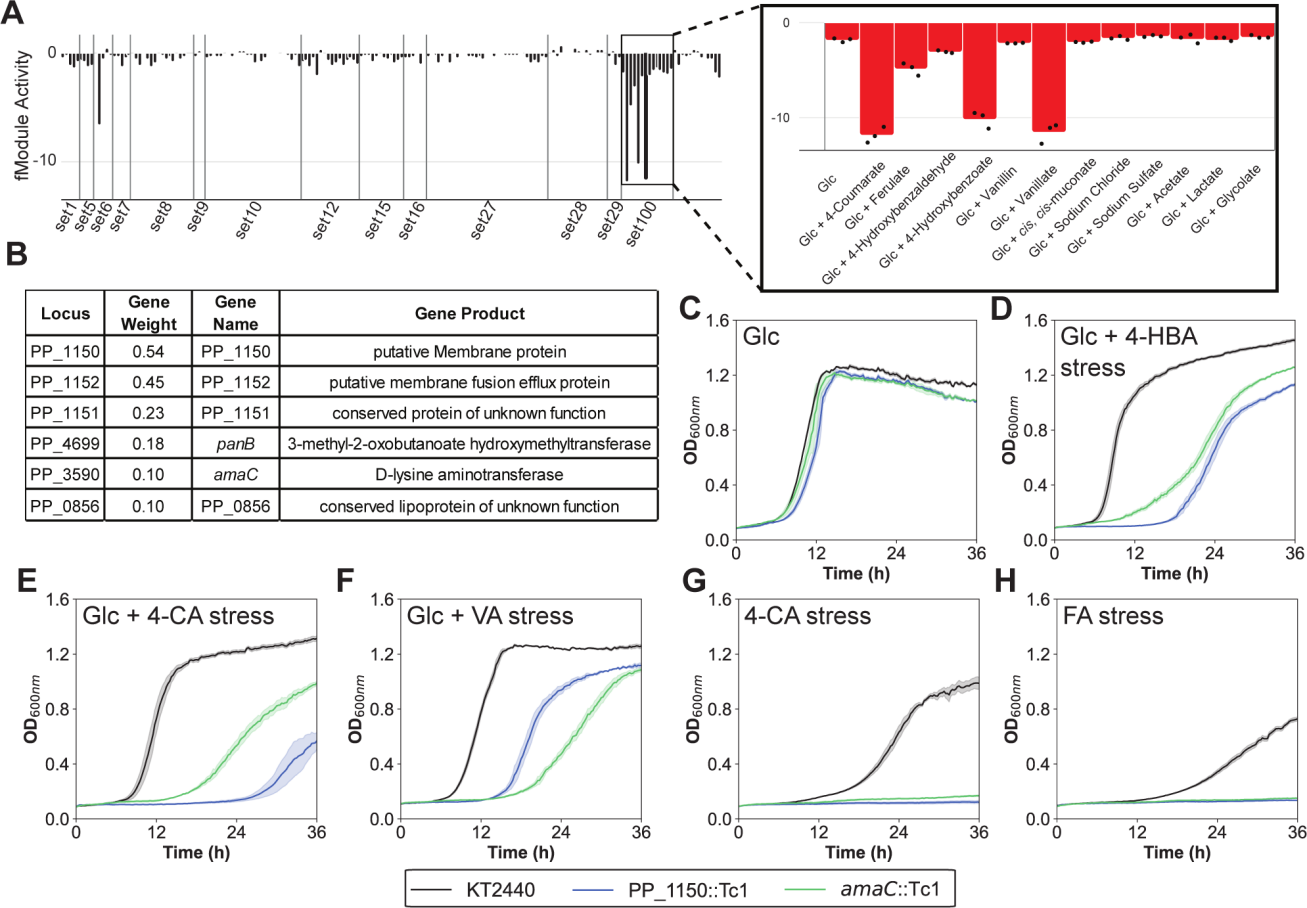
Functional knockouts of genes in fModule_35 reflect its strongly negative activity during hydroxycinnamate stress. (**A**) Activity profile of fModule_20 across all conditions in the data set, with the inset demonstrating negative activity during hydroxycinnamate catabolism and stress. (**B**) Membership and associated gene weights for fModule_20. The growth of individual transposon disruption mutants of *P. putida* (PP_1150::Tc1 and *amaC*::Tc1) was compared with wild-type (KT2440), using M9 minimal medium supplemented (**C**) 20 mM glucose as the carbon source, (**D**) 20 mM glucose with 60 mM 4-hydroxybenzoate stress, (**E**) 20 mM glucose with 60 mM 4*-*coumarate stress, (**F**) 20 mM glucose with 60 mM vanillate stress, (**G**) 100 mM 4*-*coumarate as the carbon source, or (**H**) 160 mM ferulate as the carbon source. Error shading indicates the standard deviation from the mean of three biological replicates. Glc, glucose; 4-HBA, 4-hydroxybenzoate; 4-CA, 4*-*coumarate; VA, vanillate; FA, ferulate.

Activity and gene membership of fModules also helped define pathways for tolerance to hydroxycinnamate stress. The activity of fModule_20 was strongly negative during growth on glucose with stressful concentrations of hydroxycinnamic acids such as 4-coumarate, 4-hydroxybenzoate, and vanillate (Fig. 4A). This module contained only six genes: PP_1150–1152, *amaC* (PP_3590), *panB* (PP_4699), and PP_0856 (Fig. 4B). Transposon disruption mutants of these genes were utilized for growth assays to probe the function assigned by the fModule (Fig. 4C through H). PanB is involved in the biosynthesis of pantothenate, a precursor to CoA (56, 57); therefore, this mutant was unable to grow in M9 minimal medium without supplementation of pantothenate. PP_0856 was also excluded from analysis since no transposon disruption mutants were isolated for this gene. Nonetheless, growth assays with functional knockouts of PP_1150 and *amaC* effectively recapitulated the activity trends observed for fModule_20, where both mutants grew similarly to the wild-type strain with glucose as a sole carbon source (Fig. 4C), but both exhibited growth defects during growth on glucose with high concentrations of hydroxycinnamates (Fig. 4D through F) or during growth with high concentrations of 4-coumarate or ferulate as the sole carbon and energy source (Fig. 4G and H). PP_1150–1152 constitute a membrane protein complex, so these genes may be responsible for osmotic stress mitigation. Additionally, the operon is at least partially regulated by FleQ (32, 58), and previous reports have shown that overexpression of PP_1150–1152 enables enhanced growth on high concentrations of ferulate and 4*-*coumarate, relative to wild-type *P. putida* (22). The *amaC* gene, which was also included in fModule_43 for its role in L-Phe catabolism, has not been previously identified as a fitness contributor for hydroxycinnamate tolerance, so we engineered *P. putida* to overexpress *amaC* (strains ACB272 and ACB287; Table S3). Unfortunately, *amaC* overexpression failed to improve growth with 4-coumarate, ferulate, or protocatechuate, relative to the wild-type (Fig. S10), perhaps due to regulatory mechanisms or an as-yet misunderstood contribution of this gene to the function of fModule_20.

### Inclusion of genes in multiple fModules reveals pathway integration

Notably, multiple genes were members of two fModules (70 genes), three fModules (22 genes), four fModules (nine genes), five fModules (four genes), and six fModules (two genes). Given the promiscuity of many proteins and the integrated nature of genetic networks, it is perhaps unsurprising to see several genes playing a role in multiple functionally distinct fModules. For example, *cbrB* (PP_4696) and *cysB* (PP_2327) were the two genes hat were each present in six different fModules. CbrB is a σ^54^ response regulator known to regulate central carbon metabolism and amino acid uptake (59, 60), while CysB is a LysR-type regulator that controls sulfate metabolism in *P. putida* (61). Consequently, both genes play far-reaching roles across metabolism, explaining their membership to several fModules. Overall, the ability for ICA to assign multiple functional groups to a gene better approximates the complex nature of biological systems and distinguishes this approach from other unsupervised machine learning clustering algorithms that cannot place genes into more than one group, such as *k*-means and agglomerative clustering (62, 63).

In six instances, cofactor biosynthesis genes were placed in the same fModule as genes for metabolic pathways requiring that cofactor. These are fModule_12 (biotin biosynthesis and pyruvate carboxylate) (64), fModule_13 (molybdopterin biosynthesis and benzaldehyde tolerance) (65), fModule_17 (pyrroloquinoline biosynthesis and short-chain alcohol catabolism) (23, 66), fModule_22 (cobalamin biosynthesis and ethanolamine catabolism) (67), fModule_28 (glutathione biosynthesis and vanillin catabolism/formaldehyde detoxification), and fModule_33 (biotin biosynthesis and isopentanol/isoprenol catabolism) (23).

**Fig 5:**
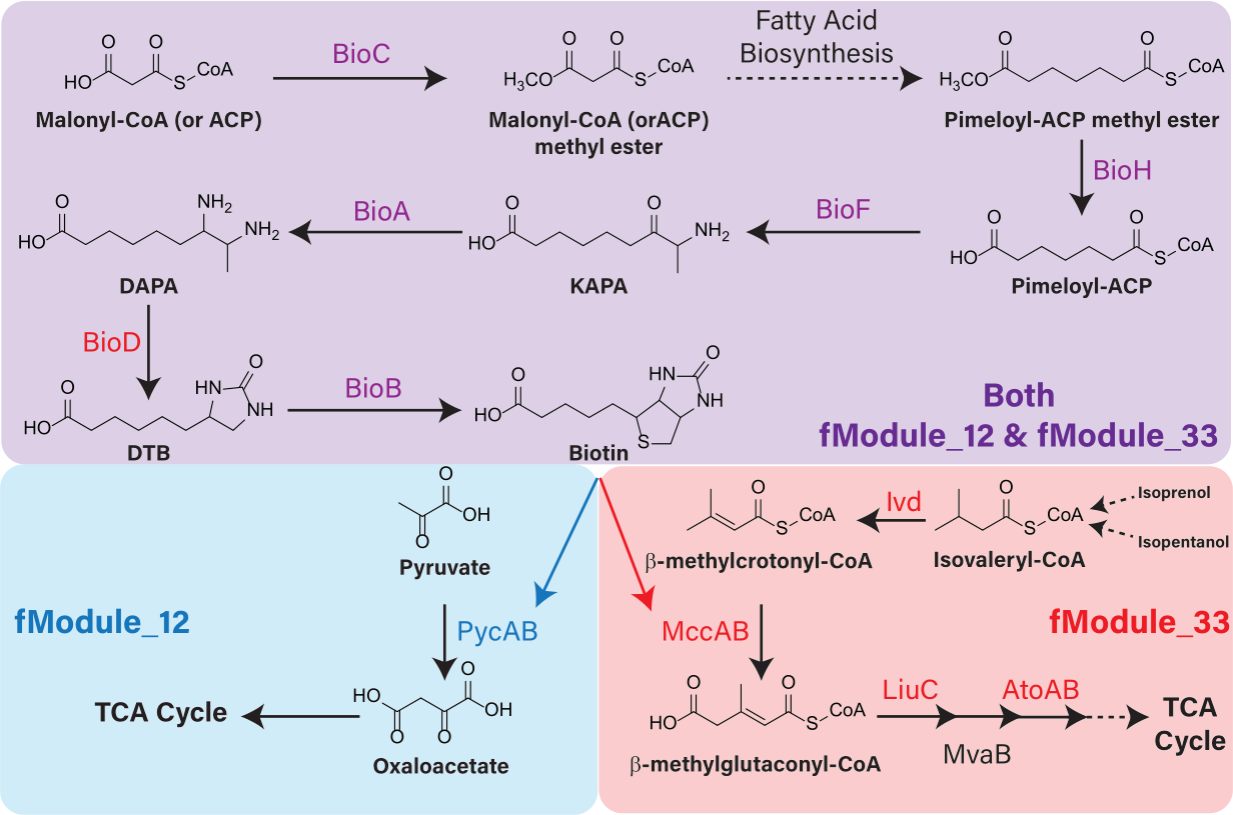
Biotin biosynthesis genes are members of both fModule_12 and fModule_33, in which each also contains genes encoding biotin-dependent carboxylases.

fModule_28 is described above, but another notable example of cofactors being grouped with enzymes dependent on those cofactors involves *bioBFHC* and the independently transcribed *bioA* gene, which encode biotin biosynthesis enzymes (68) and are members to both fModule_12 and fModule_33 (Fig. 5; File S1). fModule_12 included genes encoding the two subunits of the biotin-dependent pyruvate carboxylase, PycAB (PP_5346–5347) (64), and its regulator (PP_5348). fModule_33 included the *ivd:mccB:liuC:mccA* operon, where MccA and MccB form a biotin-binding enzyme complex (69). The *ivd:mccB:liuC:mccA* operon and the two remaining members of fModule_33, *atoAB*, are critical for the catabolism of isopentanol and isoprenol, consistent with negative activity of this fModule when isopentanol or isoprenol were used as sole carbon sources (23).

Inclusion of biotin biosynthetic pathway genes in the same fModules as those containing biotin-dependent carboxylase genes provides a powerful illustration of the physiological relationships predicted by ICA. Additionally, the presence of biotin biosynthesis genes in multiple fModules helps illustrate the potential of ICA for capturing the complex nature of biological systems, where genes and pathways can have several distinct functional relationships with other gene sets. Overall, ICA of RB-TnSeq fitness data may help identify uncharacterized links between metabolic processes and cofactor requirements.

### Single-gene fModules often contain genes with unique, far-reaching physiological roles

Interestingly, eight fModules contained only a single gene (fModules 3, 38, 39, 54, 64, 71, 81, and 82; File S1). All the single-gene fModules, excluding fModule_39, displayed negative activity across most of the conditions tested. fModule_64 contained *nadC* (PP_0787), encoding a phosphoribosyltransferase that catalyzes the third step in NAD^+^ biosynthesis from L-aspartate (70). fModule_81, contained *serA* (PP_5155), which plays a critical role in serine biosynthesis and NAD^+^/NADH recycling (71). fModule_03 contained *ridA* (PP_5303), a conserved deaminase that controls accumulation of reactive and toxic enamine and imine intermediates generated by several metabolic processes (72). fModule_71 contained *hisA* (PP_0292), a gene with a role in histidine and purine biosynthesis (73). fModule_82 contained *proI* (PP_5095), which is involved in the final step of proline biosynthesis. Surprisingly, fModule_82 also displayed negative gene fitness in conditions containing supplemented proline, suggesting that *proI* may play a role beyond proline biosynthesis (Files S1 and S2). Interestingly, fModule_38 and fModule_54 contained *ruvC* (PP_1215) and *parB* (PP_001), respectively. These genes are involved in DNA processing and repair, where ParB is a chromosome-partitioning protein and RuvC is a crossover junction endodeoxyribonuclease (74, 75). Curiously, activities for fModules 38 and 54, while generally negative across all conditions, were positive for conditions where *P. putida* was grown in a bioreactor. Overall, most single-gene fModules contained genes with known far-reaching and pleiotropic metabolic roles. Therefore, identification of single-gene fModules during the application of ICA to TnSeq data sets from other organisms may be a useful tool for identifying important genes with several connections to distinct metabolic and physiological processes.

### Comparing fModule data with iModulon data uncovers regulatory control of functional elements

**Fig 6:**
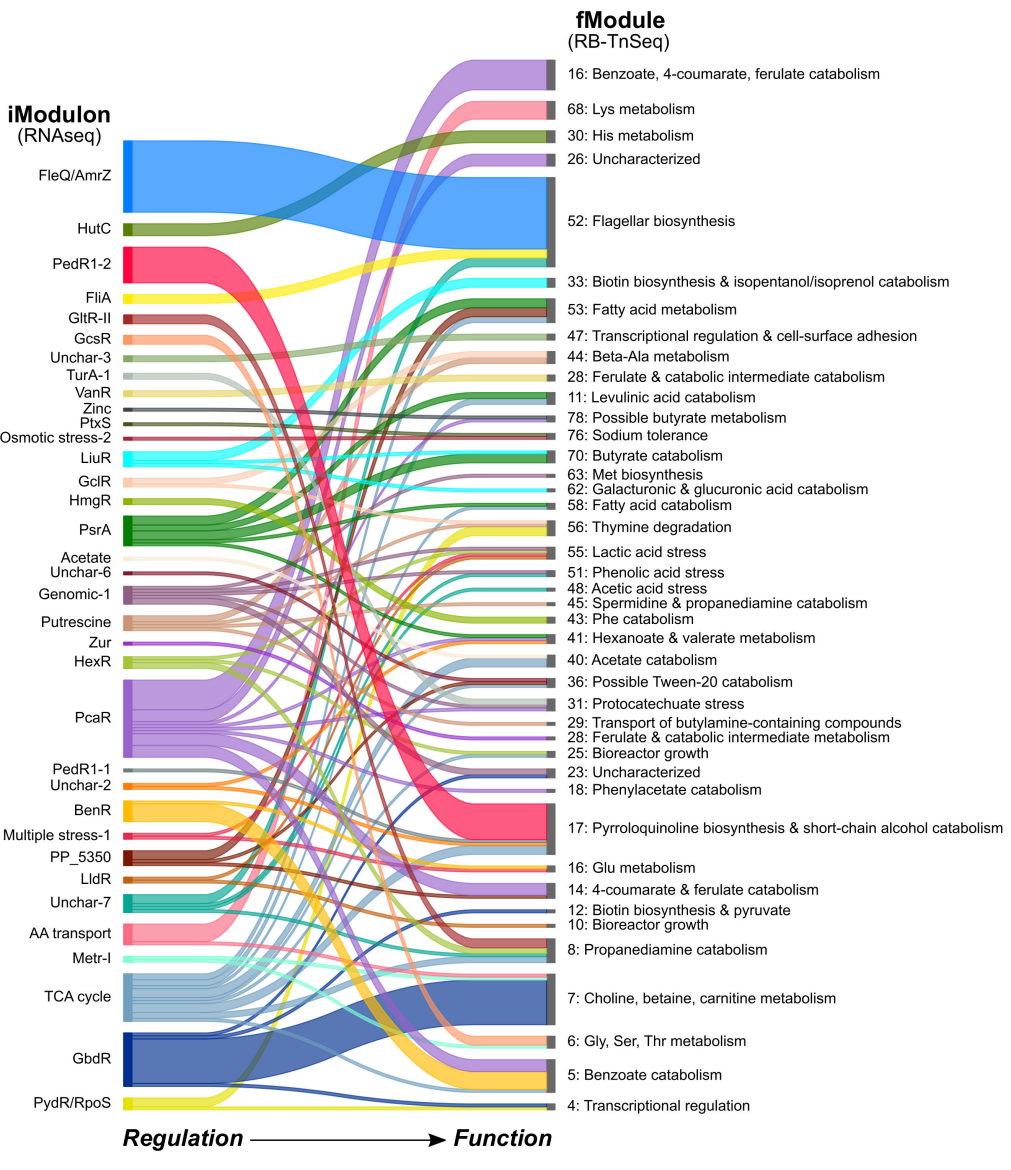
ICA assigns genes to different groups, depending on the input data set. Some KT2440 genes with membership in fModules (ICA of RB-TnSeq data, this study) were also members of iModulons (ICA of transcriptomics data, a separate study [32]). Genes that were members of the same iModulon (i.e., co-regulated) were not necessarily members of the same fModule (i.e., co-functioning). The thickness of each line corresponds to the number of genes, and labels on left and right indicate iModulon and fModule annotations, respectively. Numerical data for this figure are provided in File S2. Code for the Sankey diagram plotting function was adapted from https://github.com/anazalea/pySankey/blob/master/pysankey/sankey.py and is provided in File S3.

In a previous study, ICA was applied to transcriptomics data from *P. putida* for revealing co-regulated sets of genes, termed “iModulons” (32). The fModules described in this work are distinct from iModulons because fModules delineate groups of genes with shared function but not necessarily those with shared regulation. Nevertheless, synchronizing the expression of genes with shared physiological functions is critical to cell survival and proliferation (76). Therefore, the extent to which genes within a single fModule were colocalized within a single iModulon was explored (Fig. 6; File S2). In total, 176 genes that were grouped into an fModule were also found in one or more iModulons, and the extent to which groupings were conserved between the two analyses varied.

In some cases, genes that were members of a single iModulon were also present within a single fModule. One example of this includes the relationship between the “FleQ/AmrZ” iModulon and fModule_52 (flagellar biosynthesis), where all gene members from the “FleQ/AmrZ” iModulon with membership to an fModule belong to fModule_52 (File S2). Another example includes the “HutC” iModulon and its strong relationship to fModule_30 (His metabolism). These instances exemplify the occurrence of specific transcriptional circuits, where a transcriptional regulator, such as FleQ or HutC, controls the expression of genes all involved in a specific physiological function, such as flagellar motility or histidine metabolism, respectively (58, 77).

In other cases, gene members of a single iModulon were dispersed across several distinct, but related, fModules. As an example, several genes within fModules relevant to catabolism of specific fatty acids were members of the iModulon for PsrA, a transcriptional repressor of the β-oxidation pathway for catabolism of all fatty acids in *P. putida* (78). The “TCA cycle” iModulon offered a variation on this theme, where member genes were dispersed across several catabolic fModules that are all expected to funnel carbon toward the TCA cycle. These cases are indicative of instances where a single global transcriptional circuit may coordinate expression of related, interconnected functions.

Finally, there were instances where fModules contained genes present in two or more iModulons. Examples include fModule_5 (benzoate catabolism) and fModule_55 (lactic acid stress). In the case of fModule_5, six genes involved in the catabolism of benzoate to β-ketoadipate were included as part of the “BenR” iModulon, while four genes involved in the parallel pathway for catabolism of protocatechuate toward β-ketoadipate were members of the “PcaR” iModulon (File S2). Coordinated expression of benzoate and protocatechuate catabolic genes has been observed previously in *P. putida*, and coordination of these peripheral pathways is believed to be important for hierarchical assimilation of related metabolites that share a common downstream pathway (44, 79). Generally, these are examples of integrated transcriptional circuits, where a common cellular function is subject to multiple points of transcriptional control.

In isolation, analysis of an fModule or iModulon data set can provide high-throughput functional or regulatory information, respectively. However, comparison of the two data sets can characterize whether functional gene sets are subject to regulatory control by specific or global transcriptional circuits and determine the extent to which these circuits are integrated. Furthermore, this comparative analysis illustrates that concerted changes in fitness are not necessarily driven by concerted changes in transcriptional regulation.

In some cases, transcriptomic data may inform the function of poorly annotated fModules and vice versa. For example, fModule_26 was annotated as “Uncharacterized,” but one of its member genes belongs to the “PcaR” iModulon (File S2), which primarily contains genes required for the catabolism of aromatic compounds. Similarly, iModulon “Unchar-3” contains two genes within the “Transcriptional regulation & cell-surface adhesion” fModule (fModule_46) (File S2). Together, these data suggest that fModule_26 and the iModulon “Unchar-3”may play roles in aromatic catabolism and cell-to-surface adhesion, respectively.

Of the appropriately sized RB-TnSeq data sets that exist for other organisms, a companion iModulon data set already exists for *Escherichia coli* BW25113 (30), opening the possibility of comparing future fModule data from *E. coli* BW25113 with existing iModulon data for characterizing regulatory control of functional elements in this organism. However, for both *P. putida* and *E. coli* BW25113, the publicly available RB-TnSeq and transcriptomics data were not collected following growth in a shared set of conditions. While this did not preclude the extraction of meaningful regulatory information from the *P. putida* data sets, future studies, where gene fitness and expression data are obtained from a shared set of growth conditions, may yield stronger relationships between functional sets of genes than those achieved here.

### Conclusions

This work describes the application of ICA to a *P. putida* RB-TnSeq data set for elucidating functional relationships between genes. In total, this approach successfully (i) identified well-characterized functional relationships between sets of genes, (ii) uncovered new gene members with overlooked roles in otherwise well-characterized functional gene groups, (iii) was used to reannotate one gene’s functional role, (iv) uncovered otherwise overlooked functional gene relationships, (v) revealed instances of pathway integration, (vi) highlighted genes with far-reaching pleotropic functional roles, and (vii) was analyzed alongside iModulon data to uncover the transcriptional control mechanisms governing expression of various functional gene sets.

Overall, the technique presented in this work represents a rapid and automated approach to characterize functional modules within complex genetic networks and elucidate how an organism coordinates the expression of these modules. The approach also represents an opportunity to extract newfound value from previously generated data sets. ICA has successfully been applied to transcriptomics data sets containing as few as 23 unique conditions (80). Publicly available RB-TnSeq data sets containing at least 23 unique screening conditions exist for 37 additional organisms on the Fitness Browser (https://fit.genomics.lbl.gov/cgi-bin/myFrontPage.cgi), so it is possible that the use of these RB-TnSeq compendia might also reveal informative functional relationships between genes. Furthermore, the approach is applicable to functional genomics data sets from any organism, regardless of whether they were obtained specifically through the RB-TnSeq method. This opens ICA to a wide range of industrially, medically, or environmentally important organisms, where its ability to simultaneously identify several functional groups of genes can expedite the annotation of relatively uncharacterized organisms, as compared with classical genetic and biochemical approaches or stepwise analysis of individual data sets.

## MATERIALS AND METHODS

### Generation of an RB-TnSeq fitness data compendium for *P. putida* KT2440

An initial RB-TnSeq fitness data compendium was generated by collecting 332 publicly available *P. putida* RB-TnSeq data sets collected between 2017 and 2022, consisting of a mixture of duplicate or triplicate samples spanning 183 unique growth conditions. The Fitness Browser (https://fit.genomics.lbl.gov/cgi-bin/myFrontPage.cgi) was used to obtain data for 254/332 of the samples, and the remaining data were generated by the Beckham group, available through the NCBI Sequence Read Archive (SRA) with accession numbers PRJNA809672, PRJNA856070, and PRJNA1011287 (22, 38). In instances where gene fitness data for a particular gene did not exist across all 332 data sets, the gene was eliminated from analysis. Biological and technical replicates present in the data were not averaged or normalized in any other way prior to analysis. This resulted in a final data set, where 4,732/5,564 protein-coding genes from *P. putida* contained fitness data for the 332 samples (36). Unlike previous ICA experiments using RNAseq data (32), fitness values were not normalized by batch, since each fitness measurement was already normalized by transposon insertion counts in the baseline (“time zero”) condition. Gene fitness values, associated statistics, and metadata for each sample are available at https://github.com/beckham-lab/fModule.

### ICA of the RB-TnSeq data set

ICA was performed for the final compendium containing fitness values for 4,732 genes profiled across 332 samples, using the FastICA algorithm of the scikit-learn package (v0.23.2), as described previously (30). This algorithm was executed 100 times with random seeds and a convergence tolerance of 10^−7^. The resulting independent components (ICs) were clustered and compared using DBSCAN (81) with an epsilon of 0.1 and minimum cluster size of 50, to identify robust ICs. To account for the occurrence identical ICs with opposite signs, a distance metric (*d*) was used to compute the distance matrix as follows:


$$d_{x,y}=1-\left|\rho_{x,y}\right|$$

where *ρ*_*x,y*_ is the Pearson correlation between components *x* and *y*. The final robust ICs were then defined as the centroids of the cluster.

Since the number of selected dimensions during ICA can alter the results of the analysis (82), the optimal dimensionality was determined by comparing the number of ICs with single genes to the number of ICs correlated (Pearson *R* > 0.7) with ICs in the largest dimension, using increasing dimension from 10 to 180 and a step size of 10. The optimal dimension was chosen to be 130, in which the final number of ICs (fModules) was greater than the number of multi-gene components, while minimizing the number of single-gene ICs (Fig. S1).

Using the optimal dimensionality of 130, the member genes for each fModule were determined by iteratively removing genes with the largest gene weight size in the fModule and computing the D’Agostino K^2^ test statistic (83) for the remaining genes. All genes that were removed prior to the test statistic dropping below a cutoff value were deemed member genes of the fModule. The threshold was determined as the value for K^2^, where non-removed genes were sufficiently normally distributed around 0, as described previously (30).

### Characterization of fModules

Functional clustering annotations for each fModule were initially explored using the DAVID 2021 functional annotation clustering tool, with *P. putida* KT2440 set as the analysis background and classification stringency set to the lowest setting, keeping all other settings at default. The gene membership list for each fModule was used as input, and the DAVID functional annotation output is provided in File S1. These annotations were refined by KEGG and Cluster of Orthologous Groups (COG) information obtained using the EggNOG-mapper (84) together with fModule activity patterns across all test conditions to manually assign putative functional annotations for all fModules ( File S1). Known transcription factor associations and any iModulon membership(s) for each gene were taken from previous assignments in the study by Lim et al. (32).

The interactive *P. putida* fModuleDB page was generated by using the imodulondb_export function in the Pymodulon package, adapted from the use for the iModulonDB (85).

### Bacterial strains, plasmids, and growth conditions

Plasmids used in this study are described in Table S1, primers are listed in Table S2, bacterial strains are described in Table S3, and synthetic DNA sequences are listed in Table S4. PCR reactions were performed with Q5 High-Fidelity 2X Master Mix (New England Biolabs), and the pACB131 plasmid was assembled by the method of Gibson using NEBuilder HiFi DNA Assembly Master Mix (New England Biolabs). Plasmid-bearing *E. coli* strains were grown at 37°C and 225 rpm. *P. putida* and mutants were maintained in lysogeny broth (LB) medium at 30°C unless otherwise indicated. For overexpression of PP_3590 (*amaC*) in *P. putida* strains ACB272 and ACB287, overnight cultures of the *P. putida* KT2440 wild-type were electroporated with 500 ng of pACB127 or pACB131, respectively, according to an established method (86). Plasmids contained 1,000-bp homology arms on either side of the P_tac_:PP_3590 construct, which enabled recombination at the desired loci in the *P. putida* genome. Recombination was enabled with a previously established protocol in which transformants were selected twice on LB agar with 50 mg/L kanamycin (Km) and counter-selected twice on YT agar with 25% sucrose (87). The same procedure was used for knockout of PP_0120 (*znuA1*) in *P. putida* strain KDD007. For plasmid-based overexpression of *glcB* in *P. putida* strain ACB329, an overnight culture of the *P. putida* KT2440 wild-type was electroporated with 100 ng of pACB145, and plasmid-bearing mutants were selected on LB agar supplemented with 50 mg/L Km. All subsequent cultivations of ACB329 were performed in media supplemented with 50 mg/L Km.

### Isolation of *P. putida* strains from an individually arrayed insertion mutant library

All RB-TnSeq data in the ICA data set were generated with a previously described, randomly barcoded transposon mutant library in *P. putida* KT2440 (Putida_ML5) (88). This pooled library was individually arrayed, and barcode assignments were determined for each transposon mutant as previously described (89). Individual transposon insertion mutants (Table S3) were withdrawn from the arrayed library by scraping a small amount of relevant glycerol stock into a round-bottom tube filled with 3 mL of LB medium with 50 mg/L Km. Each mutant culture was grown overnight at 30°C and 225 rpm, and permanent stocks were generated by combining overnight culture with sterile glycerol to 20% (vol/vol). Additionally, 1 µL of overnight culture was used as a template for a PCR reaction to verify the barcode sequence of each mutant. Each PCR reaction contained 12.5 µL of Q5 High-Fidelity 2× Master Mix (New England Biolabs), 1.25 µL each of the previously described BarSeq_P1 and BarSeq_P2 primers (Table S2), 0.5 µL of dimethyl sulfoxide (Sigma-Aldrich), 1 µL of overnight culture, and water to 25 µL. Thermal cycles were as follows: (i) 98°C for 4 min; (ii) 25 cycles of 98°C for 30 s, 55°C for 30 s, and 72°C for 30 s; and (iii) 72°C for 5 min. PCR products were Sanger sequenced with oACB441 (Table S2) to verify the barcode sequence of each mutant.

Growth analysis of the *P. putida* wild-type and mutants. Growth media were prepared by mixing equal volumes of 2× M9 medium and 2× carbon source solution to achieve a final concentration of 1× M9 medium (6.78 g/L Na_2_HPO_4_, 3 g/L KH_2_PO_4_, 0.5 g/L NaCl, 1 g/L NH_4_Cl, 2 mM MgSO_4_, 100 µM CaCl_2_, and 18 µM FeSO_4_) with the desired final concentration of each carbon source. For zinc experiments with strain KDD007, 100 nM ZnSO_4_ was added to 1× M9 medium. Carbon sources were glucose, 4-hydroxybenzoate, 4-coumarate, ferulate, vanillate, protocatechuate, vanillin, benzoate, and butanol. Where indicated, M9 medium omitted NH_4_Cl and instead utilized an alternative nitrogen source: 5 mM of L-Glu, L-Phe, L-Tyr, or D-Lys. Aromatic compounds were titrated with base (4 M NaOH) to solubilize the compound in aqueous solution prior to sterile filtration and addition to media. Amino acid nitrogen sources were titrated with acid (1 M H_2_SO_4_, for L-Tyr) or base (4 M NaOH, for D-Lys and L-Phe) to solubilize the compound in aqueous solution prior to sterile filtration and addition to media. The pH of solubilized L-Tyr was 3.8, but upon addition of this solution to M9 medium, the pH remained neutral (pH = 7.1), as with all other carbon and nitrogen sources. No precipitation of carbon or nitrogen sources was observed during growth. All chemicals for media preparation were obtained from Sigma-Aldrich, except for protocatechuate (Acros Organics) and D-Lys (Ambeed Inc.).

To assess the growth of the *P. putida* wild-type and all mutants, besides Δ*znuA*1, biological triplicate cultures of each strain were inoculated from single colonies into 4 mL of LB medium in round-bottom tubes and incubated overnight at 30°C and 225 rpm. For each condition, 2 µL of overnight culture was directly inoculated into 200 µL of medium (1:100 dilution) in a Honeycomb plate (Growth Curves Ltd.). Plates were incubated at 30°C and maximum shaking speed in a BioscreenC Pro instrument (Growth Curves Ltd.), and the optical density at 600 nm (OD_600_) was measured every 15 min.

For growth experiments with zinc added to the medium, biological triplicate cultures of the *P. putida* wild type and KDD007 were inoculated from single colonies into 4 mL of LB medium in round-bottom tubes and incubated overnight at 30°C and 225 rpm. The next day, overnight cultures were used to inoculate secondary seed cultures in 5 mL M9 medium + 30 mM glucose. Seed cultures were grown to mid-log phase (~4 h at 30°C and 225 rpm), and then, each culture was washed twice in 1× M9 salts and diluted to an OD_600_ of 3. Next, 10 µL of each cell suspension was directly inoculated into wells of a Nunc Edge 2.0 96-well plate (Thermo Scientific) containing 200 µL of M9 medium with 30 mM glucose or 20–80 mM vanillate as the carbon source. In half of the samples, media were amended to include 100 nM ZnSO_4_. Plates were incubated at 30°C and 500 rpm in a LogPhase 600 instrument (Agilent), and the OD_600_ was measured every 20 min. Background correction for each sample was performed by subtracting the average optical density of the appropriate negative controls.

## Data Availability

Sequence data from RB-TnSeq data sets 100 and 101 are available at the NCBI SRA, with accession numbers PRJNA809672, PRJNA856070, and PRJNA1011287 (22, 38). Perl and Python scripts used for analysis of data sets 100 and 101 are accessible from https://github.com/beckham-lab/RB-TnSeq.git. All remaining fitness data are available at https://fit.genomics.lbl.gov/. Source code for fModule analysis and figures may be found at https://github.com/fModules/putida-code, which contains the Jupyter notebook files used for analysis as well as raw input and output data. The interactive website, containing activity and gene information for each fModule, can be found at: https://fmodules.github.io/putida. Gene fitness values, associated statistics, and metadata for each sample are available at https://github.com/beckham-lab/fModule. Python code for the Sankey diagram plotting function was adapted from https://github.com/anazalea/pySankey/blob/master/pysankey/sankey.py and is provided in File S3.

## SUPPLEMENTAL MATERIAL

File S1 msystems.00942-23-s0001.xlsx Functional annotation clustering for fModules.

File S2 msystems.00942-23-s0002.xlsx Gene names and weights in fModules.

File S3 msystems.00942-23-s0003.txt Code for generating Sankey diagram in Figure 6.

Supplemental Materials msystems.00942-23-s0004.pdf Figures S1-S10; Tables S1-S4.

ASM does not own the copyrights to Supplemental Material that may be linked to, or accessed through, an article. The authors have granted ASM a non-exclusive, world-wide license to publish the Supplemental Material files. Please contact the corresponding author directly for reuse.
