# Supplementary material for: Machine learning analysis of RB-TnSeq fitness data predicts functional gene modules in Pseudomonas putida KT2440
Source: mSystems. 2024 Feb 7;9(3):e00942-23. doi: 10.1128/msystems.00942-23 (PMC10949508; doi:10.1128/msystems.00942-23)
Supplement: Supplemental Materials — Figures S1-S10; Tables S1-S4. [file msystems.00942-23-s0004.pdf]

Supplementary Information for:

Machine learning analysis of RB-TnSeq fitness data predicts functional gene modules in *Pseudomonas putida* KT2440

Andrew J. Borchert, Alissa C. Bleem, Hyun Gyu Lim, Kevin Rychel, Keven D. Dooley, Zoe A. Kellermyer, Tracy L. Hodges, Bernhard O. Palsson, and Gregg T. Beckham

Contents

Figure S1.......... 2
Figure S2.......... 3
Figure S3.......... 4
Figure S4.......... 5
Figure S5.......... 6
Figure S6.......... 7
Figure S7.......... 8
Figure S8.......... 10
Figure S9.......... 11
Figure S10.......... 11
Table S1 12
Table S2 144
Table S3 17
Table S4 19
References.......... 20

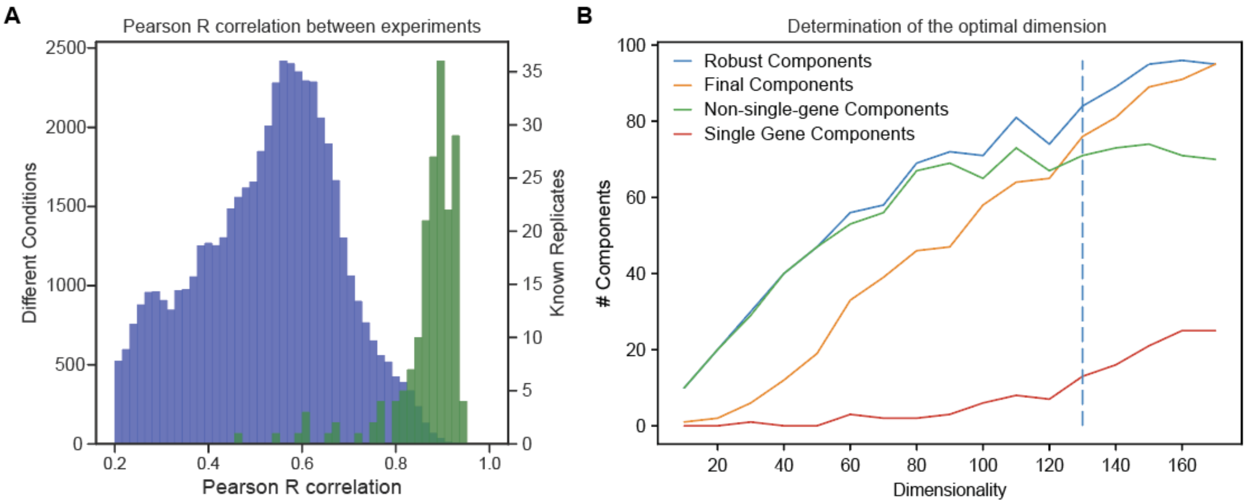

Figure S1. Dataset replicate quality and ICA result. (A) Pearson R correlation coefficients between every pair of the 332 experiments. Green and blue bars indicate a pair of known replicate samples and non-replicate samples (different conditions). **(B)** Determination of the optimal ICA dimension for the dataset. The optimal dimension was chosen where the number of final components is equal or greater than the number of non-single gene components.

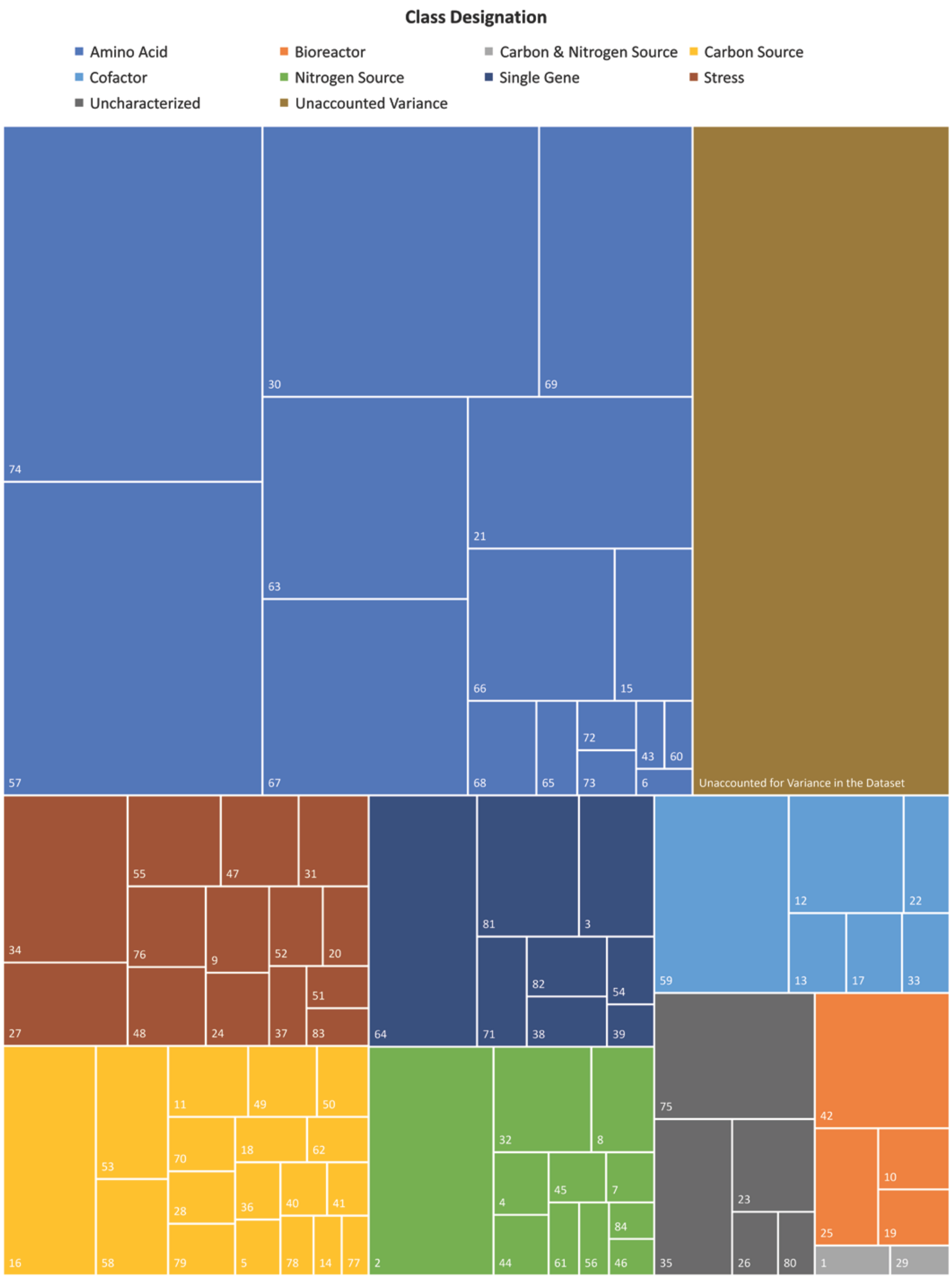

Figure S2. Tree map depicting the proportion of variance explained by each fModule. The proportion of variance explained by each fModule corresponds to its area and the color of each fModule corresponds to a functional class designation. Numbers in the lower left of each box indicate the fModule number, as provided in Table 1.

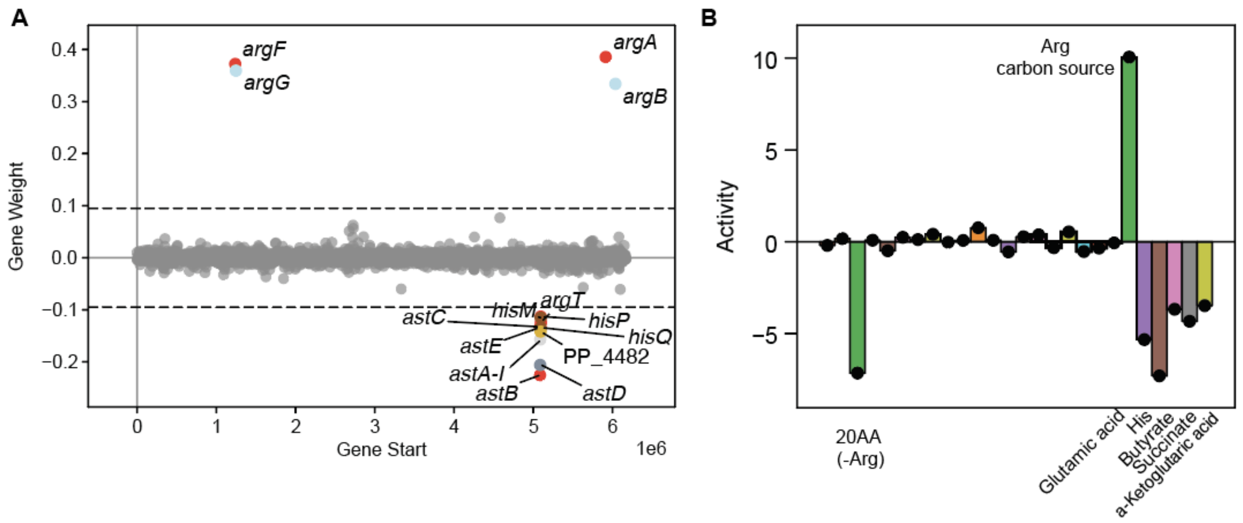

Figure S3. Characteristics of fModule_21 indicate its member genes function together in L-arginine metabolism. (A) Gene weights and **(B)** activity of fModule_21 under experimental conditions lacking L-Arg supplementation.

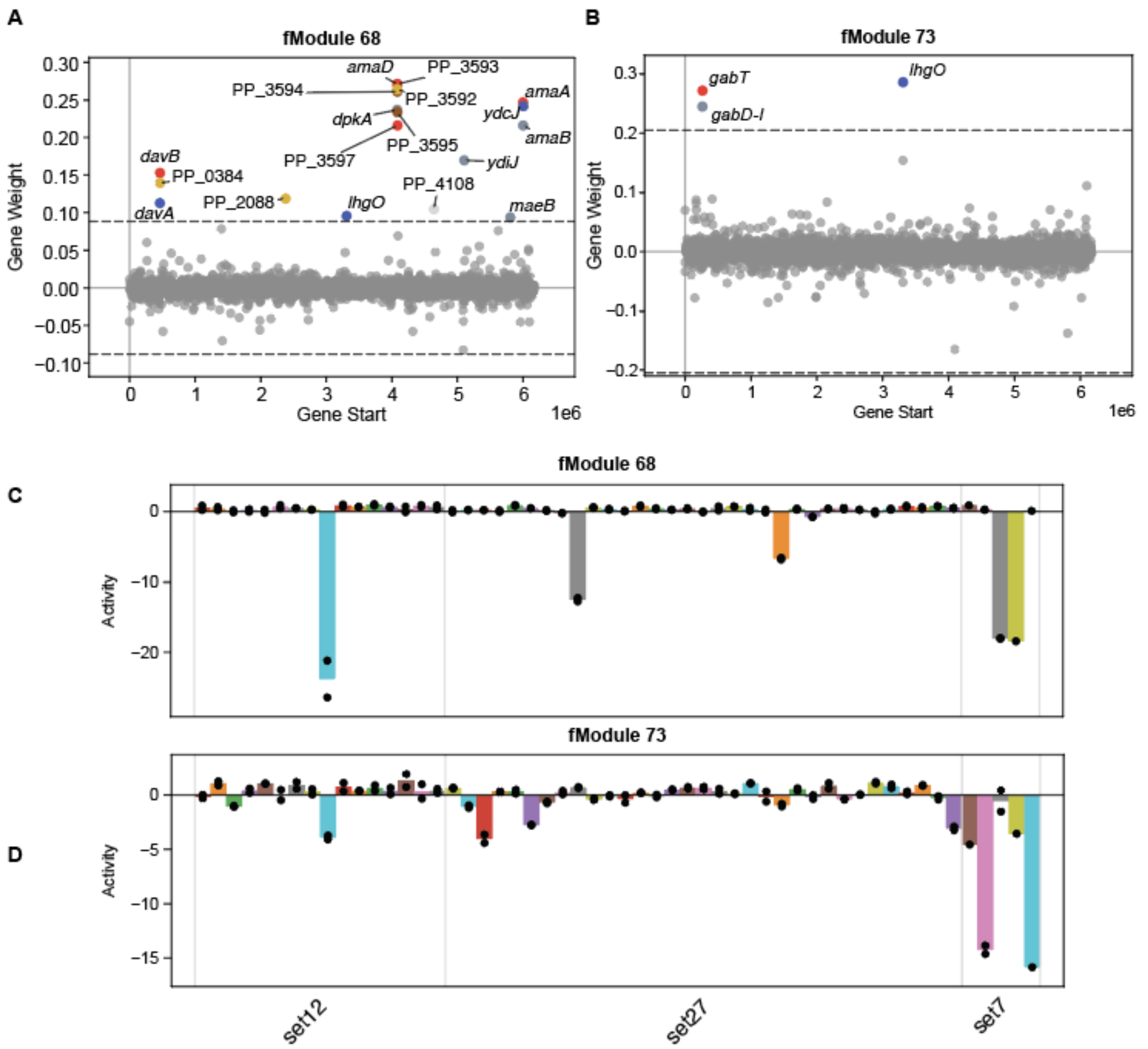

Figure S4. Activities of fModule_68 and fModule_73 indicate a role for member genes in lysine regulation, transport, and catabolism. (A,B) Gene weights for fModule_68 and fModule_73, respectively. **(C,D)** Activity of fModule_68 and fModule_73, respectively, when L-lysine, D-lysine, or catabolic intermediates were used as a carbon or nitrogen sources.

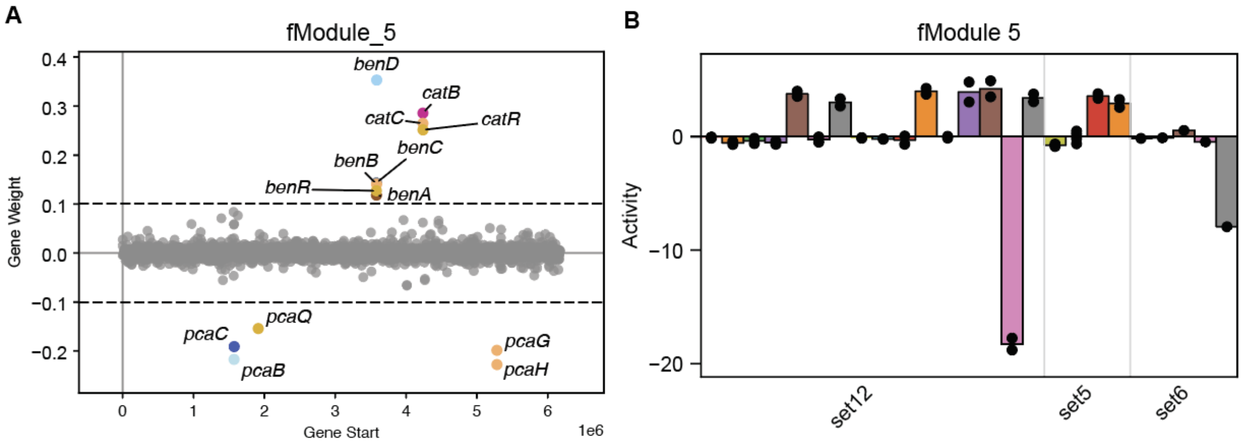

Figure S5. Benzoate catabolism is the indicated function of fModule_5. ICA grouped genes with well-characterized, shared roles in benzoate catabolism, as indicated by **(A)** gene weights and **(B)** fModule activity values in conditions where benzoate was provided as a sole carbon source.

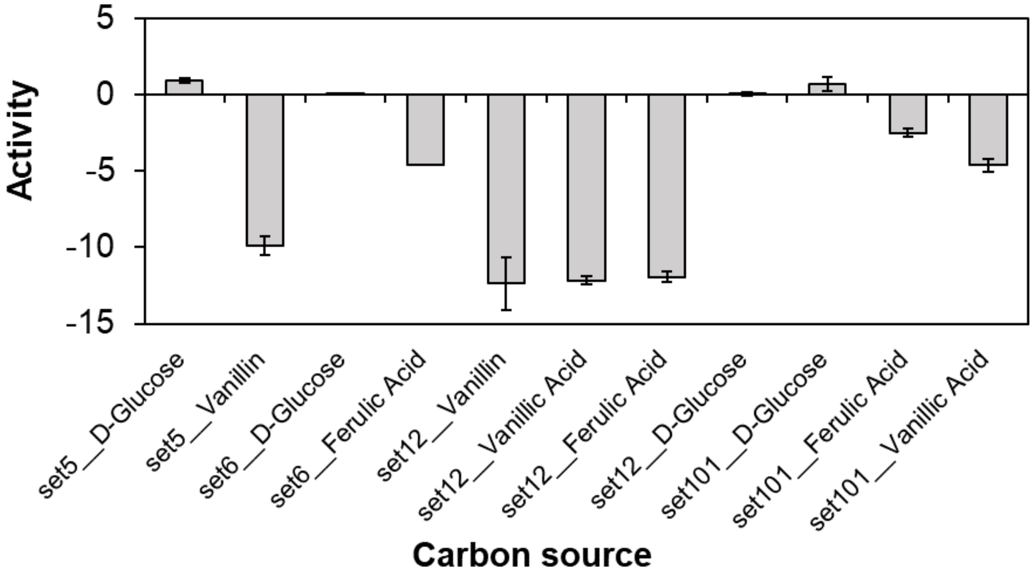

Figure S6. Activity of fModule_28 in conditions where *O*-methoxylated aromatics were provided as a sole source of carbon. Each condition on the x-axis refers to growth on a single carbon source, and the set number indicates the RB-TnSeq experiment set from which fitness data were collected. Error bars represent the standard deviation from the mean of 2-3 replicate RB-TnSeq experiments.

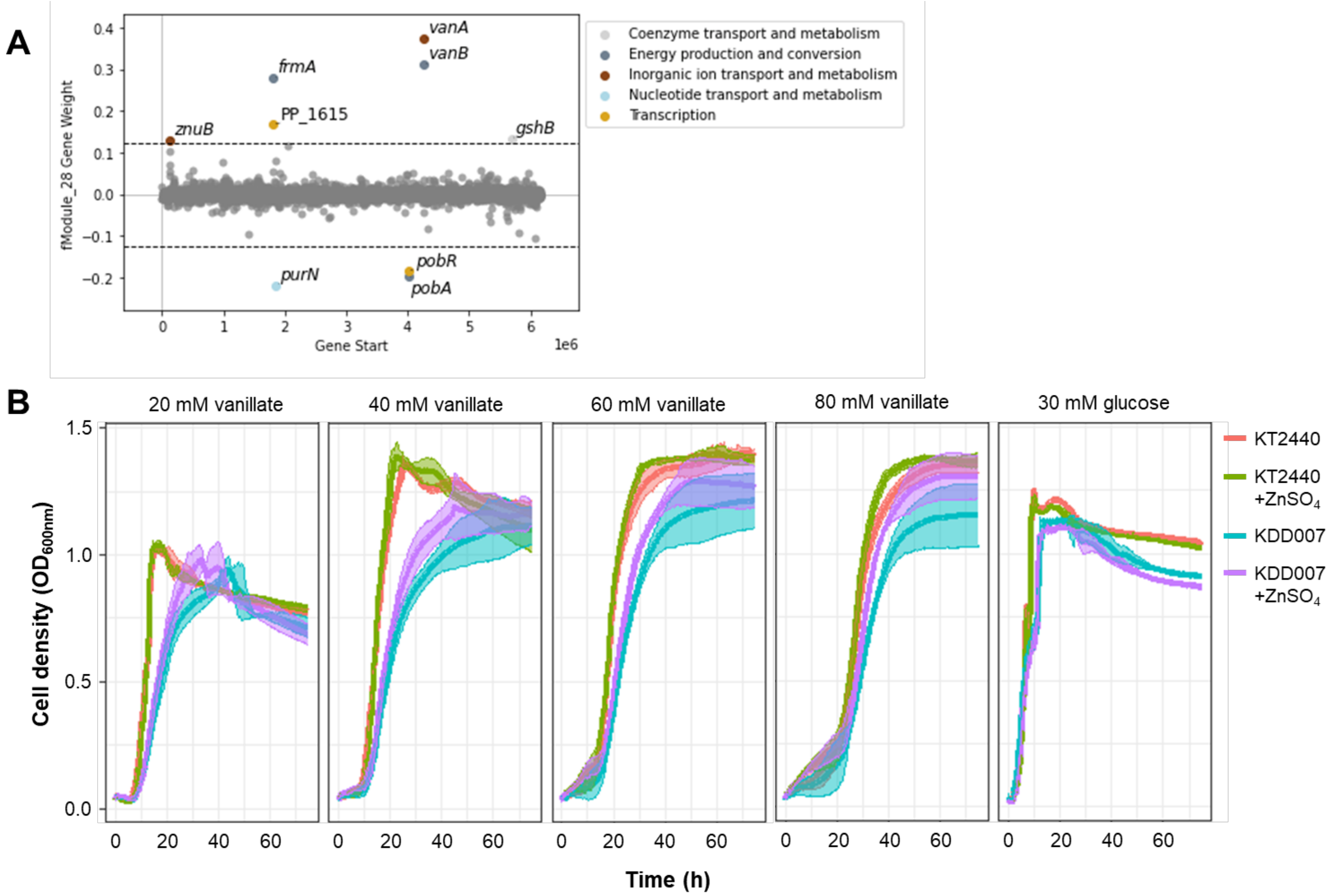

Figure S7. Zinc transport is important for growth with vanillate. (A) Members of fModule_28 have previously described roles in demethylation and formaldehyde tolerance. The *znuB* gene, which encodes the inner membrane pore of a zinc ABC transporter, was also included in fModule_28. (B) For *P. putida* KT2440 wild-type (KT2440), addition of 100 nM $ZnSO_4$ to the growth medium resulted in a slight improvement in growth with vanillate, but not glucose, as the carbon and energy source. A *P. putida* mutant impaired in zinc transport (KDD007, Δ*znuA1*) displayed growth defects relative to wild-type in all conditions, but the effect was more pronounced during growth with vanillate as the carbon and energy source. Addition of 100 nM $ZnSO_4$ to vanillate growth media resulted in a slight improvement in growth for KDD007, likely due to the presence of multiple zinc transporters in *P. putida* (1). Error shading indicates the standard deviation from the mean of three biological replicates.

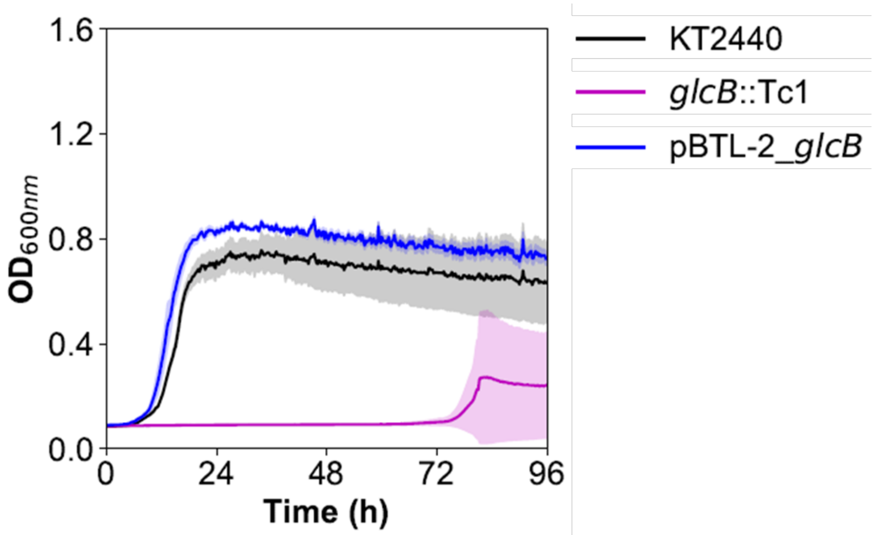

Figure S8. Disruption of *glcB* leads to very poor growth on ferulate. Growth of the *glcB* transposon disruption mutant (*glcB*::Tc1) was compared to wild-type (KT2440) and an overexpression strain (pBTL-2_*glcB*) in M9 minimal medium with 10 mM ferulate.

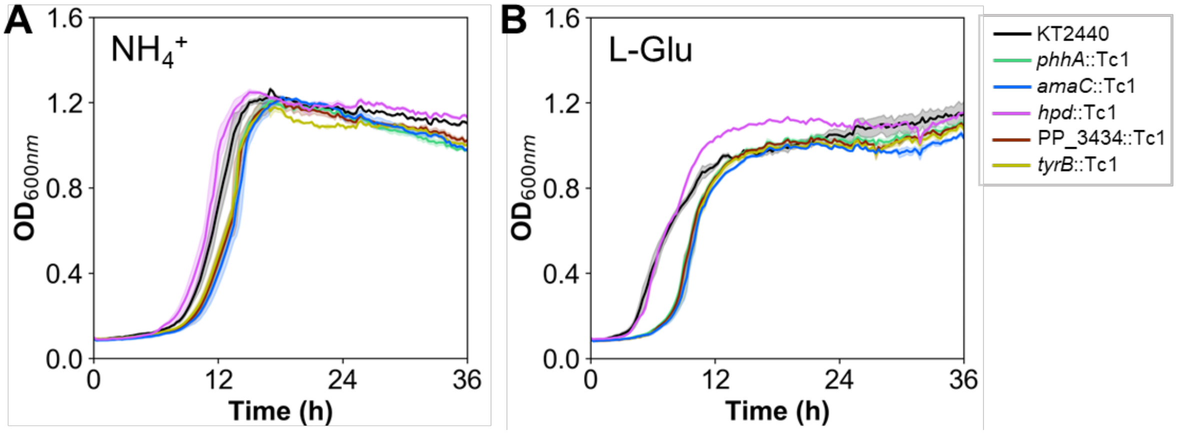

Figure S9. Growth with L-Glu as the sole nitrogen source. Growth of individual transposon disruption mutants (gene::Tc1) was compared to wild-type (KT2440) in M9 minimal medium in microtiter plates, using 20 mM glucose as a carbon source and **(A)** 5 mM ammonium or **(B)** 5 mM L-Glu as the nitrogen source. Error shading indicates the standard deviation from the mean of three biological replicates.

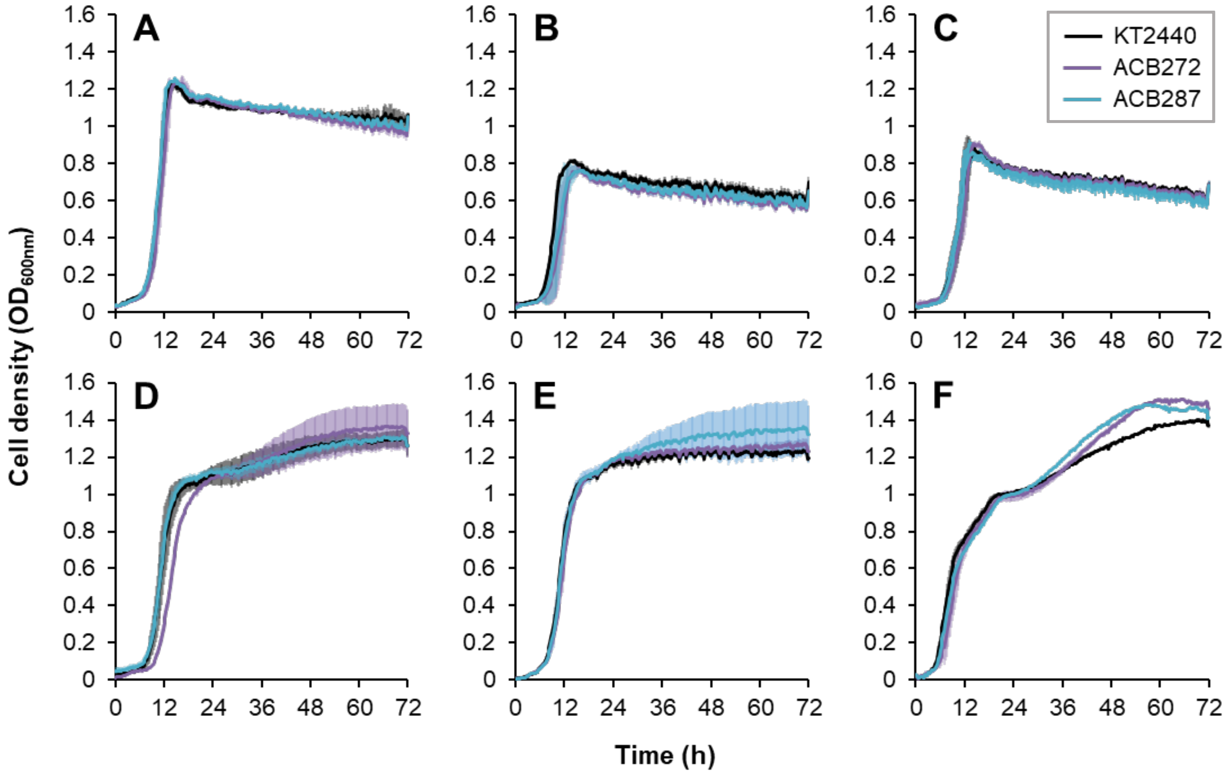

Figure S10. Overexpression of *amaC* does not improve growth with or stress tolerance to aromatics, relative to wild-type. The growth of *P. putida* wild-type (KT2440) was compared to two strains engineered for overexpression of *amaC*. Strain ACB272 contains the strong P_{tac} promoter upstream of the native *amaC* sequence (P_{tac}:*amaC*), while strain ACB287 contains a second copy of *amaC* under control of the P_{tac} promoter at the *fpvA* locus (*fpvA*:P_{tac}:*amaC*). All strains were cultivated in M9 minimal medium in microtiter plates with **(A)** 20 mM glucose, **(B)** 10 mM 4-coumarate, **(C)** 10 mM ferulate, **(D)** 20 mM glucose + 60 mM 4-coumarate, **(E)** 20 mM glucose + 60 mM ferulate, or **(F)** 20 mM glucose + 60 mM protocatechuate. Error bars indicate the standard deviation from the mean of three biological replicates.

Table S1. Plasmids used in this study.

Plasmid	Description	Construction details
**pK18sB**	Sucrose counter-selection allelic exchange vector for *P. putida* KT2440; Km^r	GenBank: MH166772.1
**pBTL-2**	Broad host range vector (replicative); Km^r	GenBank: DQ058740.1
**pACB127**	pK18sB-based suicide vector for integration of the P_{tac} promoter upstream of the native *amaC* (PP_3590) CDS in *P. putida* KT2440.	Insert (see **Table S12**) was synthesized by Twist Biosciences and cloned into the EcoRI/HindIII insertion sites of pK18sB by Twist Biosciences.
**pACB131**	pK18sB-based suicide vector for integration of a second copy of *amaC* (PP_3590), under control of P_{tac} promoter, into fpvA locus in *P. putida* KT2440.	The pK18sB vector backbone, including the P_{tac} promoter and tonB terminator, was amplified from the same template as pACB085 (2) using oZK027 and oZK028. PP_3590 was amplified from KT2440 genomic DNA using oZK025 and oZK026. The ribosome binding site, designed using the Salis tool for RBS design (3), was introduced using the primers oZK025 and oZK028. The vector backbone and PP_3590 were assembled via Gibson assembly and transformed into chemically competent *E. coli* DH5α F'Iq cells. Transformed colonies underwent colony PCR with oCJ308 and oCJ309. A correct colony was miniprepped and Sanger sequenced (Azenta) with oCJ925, oZK029, and oZK030 to verify the correct sequence.
**pACB145**	pBTL-2 vector for overexpression of *glcB* (PP_0356) under control of the P_{lac} promoter in KT2440.	pBTL-2 underwent restriction digest with EcoRV and XbaI (New England Biolabs), and the 2586 bp band was isolated by gel excision and column clean-up (Macherey-Nagel). The *glcB* gene and its native RBS sequence were amplified from KT2440 genomic DNA using oACB516 and oACB517 to generate a 758 bp product. The vector backbone and *glcB* insert were assembled via Gibson assembly and transformed into chemically competent *E. coli* DH5α F'Iq cells. Transformed colonies underwent colony PCR with oCJ162 and oCJ163. A correct colony was miniprepped and Nanopore sequenced (Plasmidsaurus) to verify the correct sequence.

**pKDD001**	pK18sb-based suicide vector for deletion of *znuA1* (PP_0120) in KT2440.	The pK18sB vector backbone was amplified using the oKDD005/006 primer pair. Genomic regions 1000bp upstream and downstream of *znuA1* were amplified using oKDD001/002 and oKDD003/004, respectively. These amplicons were then assembled via Gibson assembly and transformed into chemically competent *E. coli* DH5α F'Iq cells. Transformed colonies underwent colony PCR with oCJ680 and oCJ547. A correct colony was miniprepped and Nanopore sequenced (Plasmidsaurus) to verify the correct sequence.

[a]Km^r, resistance to kanamycin.

Table S2. Sequences of DNA oligos used in this study. Integrated DNA Technologies (IDT) was used for synthesis.

Primer	Sequence (5'→3')	Description
**oZK010**	CACACTTCCATCCATAGC	Diagnostic: binds 5' of *amaC* to verify genomic integration of P_{tac}:*amaC* in ACB272. Forward.
**oZK011**	CGGTGTAGCTGAACATGC	Diagnostic: binds 3' of *amaC* to verify genomic integration of P_{tac}:*amaC* in ACB272. Reverse.
**oZK012**	GCAGTTGATCATCGTCATCG	Sequencing: binds 5' of *amaC* to verify genomic integration of P_{tac}:*amaC* in ACB272. Forward.
**oZK025**	CCATAGCCCTATATTCTCAAG GAGTATTTTGTGTTCAAACAT GTCGATGCC	Amplifies PP_3590 from KT2440 genomic DNA and adds ribosome binding site for assembly into pACB131. Forward.
**oZK026**	GGTCGGAGGCTTTTGACTTTA CTTCTGAACGGCAGCGAAC	Amplifies PP_3590 from KT2440 genomic DNA for assembly into pACB131. Reverse.
**oZK027**	AAGTCAAAAGCCTCCGACC	Amplifies pK18sB vector backbone from pACB101 for assembly into pACB131. Forward.
**oZK028**	CTCCTTGAGAATATAGGGCTA TGGATGGAAGTGTGAAATTGT TATCCGCTC	Amplifies pK18sB vector backbone from pACB101 for assembly and adds ribosome binding site for assembly into pACB131. Reverse.
**oZK029**	GTCTGGGTCAGCAACCCG	Sequencing: binds in PP_3590 for sequencing of pACB131. Also used to verify genomic integration of P_{tac}:PP_3590 in ACB287. Forward.
**oZK030**	GAGTGACCCATGACTTCATG	Sequencing: binds towards 3' end of tonB terminator for sequencing of pACB131. Reverse.
**oZK031**	GACAAGAAGTACTACCAGGGC	Sequencing: binds towards 5' end of P_{tac}. Forward.
**oZK032**	CGGCGTACTGAGCGATGCTG	Sequencing: binds towards 3' end of PP_3590. Forward.
**oCJ162**	CCCAGGCTTTACACTTTATGC	Diagnostic: binds 5' of insert in pBTL-2 to verify assembly of pACB145. Forward.
**oCJ163**	TTGTCCAGCAGGGTTGTC	Diagnostic: binds 3' of insert in pBTL-2 to verify assembly of pACB145. Reverse.
**oCJ479**	GTTTCACTTGATGCTCGATG	Binds 5' of P_{lac} in pACB145; used for sequencing. Forward.
**oCJ308**	ACATCACCTGCTACGAAG	Diagnostic: binds in *fpvA* to check assembly of pACB131. Also used to

		verify genomic integration of P_{tac}:PP_3590 in ACB287. Forward.
**oCJ309**	GATTCGCATGACACGCTG	Diagnostic: binds downstream of PP_3590 and tonB terminator to check assembly of pACB131. Also used to verify genomic integration of P_{tac}:PP_3590 in ACB287. Reverse.
**oCJ312**	CACGCCTGCTTCATTGAAC	Diagnostic: binds at 3' end of PP_4219 to verify genomic integration of P_{tac}:PP_3590 in ACB287. Reverse.
**oCJ925**	GCCAAGTACAGCTTCTGATC	Sequencing: binds towards 5' end of P_{tac} promoter for sequencing of pACB131. Forward.
**BarSeq_P1**	AATGATACGGCGACCACCGAG ATCTACACTCTTTCCCTACAC GACGCTCTTCCGATCTNNNNN GTCGACCTGCAGCGTACG	For barcode verification in individual transposon mutants. Previously described (4).
**BarSeq_P2**	CAAGCAGAAGACGGCATACGA GATCGTGATGTGACTGGAGTT CAGACGTGTGCTCTTCCGATC TGATGTCCACGAGGTCTCT	For barcode verification in individual transposon mutants. Previously described (4).
**oACB441**	GACCACCGAGATCTACAC	For Sanger sequencing of amplified barcodes from individual transposon insertion mutants.
**oACB516**	GCGGATAACAATTTCACACTC TGTCTGGCAGGCCGCAC	Amplifies *glcB* (PP_0356) and its native RBS region from KT2440 for Gibson assembly into pBTL-2 to generate pACB145. Forward.
**oACB517**	TGAGGCTCGTCCTGAATGATT TACAACCCGTTACGCGCCTTG AACTC	Amplifies *glcB* (PP_0356) and its native RBS region from KT2440 for Gibson assembly into pBTL-2 to generate pACB145. Reverse.
**oKDD001**	tgacatgattacgaattcgCC TTGTACCGGTTCGTCGA	Amplifies 1000bp upstream region of *znuA1* (PP_0120) from KT2440 for Gibson assembly into pK18sB to generate pKDD001. Forward.
**oKDD002**	gccgttcgcgctgtagacGGT GGATACTCGAATCTTCAGAA	Amplifies 1000bp upstream region of *znuA1* (PP_0120) from KT2440 for Gibson assembly into pK18sB to generate pKDD001. Reverse.
**oKDD003**	aagattcgagtatccaccGTC TACAGCGCGAACGGC	Amplifies 1000bp downstream region of *znuA1* (PP_0120) from KT2440 for Gibson assembly into pK18sB to generate pKDD001. Forward.
**oKDD004**	acggccagtgccaagcttATG TCAGTCTTCACCCCCGT	Amplifies 1000bp downstream region of *znuA1* (PP_0120) from KT2440 for Gibson assembly into pK18sB to generate pKDD001. Reverse.

**oKDD005**	gggggtgaagactgacataagcttggcACTGGCCGTCGTTTTACAA	Amplifies pK18sB vector backbone for Gibson assembly of pKDD001. Forward.
**oKDD006**	cgacgaaccggtacaaggcgaattcgtaATCATGTCATAGCTGTTTCCTGT	Amplifies pK18sB vector backbone for Gibson assembly of pKDD001. Reverse.
**oKDD007**	TGTACGCAATGGGAGTGGTC	Diagnostic: Binds upstream of *znuA1*. Used to confirm removal of *znuA1* from KT2440 genome. Forward.
**oKDD008**	GCCGGGATGGATGTGATGAT	Diagnostic: Binds downstream of *znuA1*. Used to confirm removal of *znuA1* from KT2440 genome. Reverse.
**oCJ680**	TTTGTGATGCTCGTCAGGG	Diagnostic: Used to confirm correct insertion of the *znuA1* flanking regions into the pK18sb vector backbone. Forward.
**oCJ547**	CCATCTTGTTCAATCATGCG	Diagnostic: Used to confirm correct insertion of the *znuA1* flanking regions into the pK18sb vector backbone. Forward.

Table S3. Bacteria used in this study, including construction details for engineered strains and barcode details for individually arrayed mutants.

Strain	Genotype	Details[a]
**KT2440**	*Pseudomonas putida* KT2440 wild-type	Wild-type strain, ATCC 47054. Cm^r Amp^r
**ACB228**	*P. putida* KT2440 PP_1150:Tc1	From individually arrayed transposon mutant library. KT2440 PP_1150::$Tc_{1317952}$(Km)[b]
**ACB265**	*P. putida* KT2440 *glcB*:Tc1	From individually arrayed transposon mutant library. KT2440 PP_0356::$Tc_{1434721}$(Km)[b]
**ACB266**	*P. putida* KT2440 *amaC*:Tc1	From individually arrayed transposon mutant library. KT2440 PP_3590::$Tc1_{4080328}$(Km)[b]
**ACB272**	*P. putida* KT2440 P_{tac}:*amaC*	*P. putida* KT2440 electrocompetent cells were transformed with 500 ng pACB127. Colonies were selected twice on LB + 50 mg/L kanamycin agar and twice on YT + 25% sucrose agar. The counterselected colonies underwent colony PCR with oZK010 and oZK011 to verify insertion of the TonB terminator, P_{tac} promoter, and ribosome binding site upstream of the native *amaC* (PP_3590) locus. A colony with the correct 1098 bp insertion band was amplified again with oZK011 and oZK012, and the purified PCR product was sequenced with oZK012 to verify the genomic sequence
**ACB280**	*P. putida* KT2440 *hpd*:Tc1	From individually arrayed transposon mutant library. KT2440 PP_3433::$Tc_{13890514}$(Km)[b]
**ACB286**	*E. coli* DH5α F'I^q	Plasmid-bearing strain containing pACB131. Km^R.
**ACB287**	*P. putida* KT2440 *fpvA*:P_{tac}:*amaC*	*P. putida* KT2440 electrocompetent cells were transformed with 500 ng pACB131. Colonies were selected twice on LB + 50 mg/L kanamycin agar and twice on YT + 25% sucrose agar. The counterselected colonies underwent colony PCR with oZK029 and oCJ312 to verify insertion of the P_{tac} promoter, ribosome binding site, *amaC* (PP_3590), and the TonB terminator at the *fpvA* locus. A colony with the correct 1908 bp insertion band was amplified again with oCJ308 and oCJ309, and the purified PCR product was sequenced with oZK031, oZK029, oZK026, and oZK032 to verify the genomic sequence.
**ACB327**	*E. coli* DH5α F'I^q	Plasmid-bearing strain containing pACB145. Km^R.
**ACB329**	*P. putida* KT2440 + pACB145	Plasmid-bearing strain containing pACB145. *P. putida* KT2440 wild-type electrocompetent cells were transformed with 100 ng pACB145. Three transformants were pooled into a single culture to generate the strain stock. Km^R.

**AJB221**	*phhA*:Tc1	From individually arrayed transposon mutant library. KT2440 *phhA*::$Tc1_{5102513}$(Km)[b]
**AJB222**	PP_3434:Tc1	From individually arrayed transposon mutant library. KT2440 PP_3434::$Tc1_{3890852}$(Km)[b]
**AJB224**	*tyrB*:Tc1	From individually arrayed transposon mutant library. KT2440 *tyrB*::$Tc_{12233960}$(Km)[b]
**AJB223**	*panB:*Tc1	From individually arrayed transposon mutant library. KT2440 *panB*::$Tc1_{5341635}$(Km)[b]
**KDD006**	*E. coli* DH5α F'I^q	Plasmid-bearing strain containing pKDD001. Km^R.
**KDD007**	*P. putida* KT2440 Δ*znuA1*	*P. putida* KT2440 electrocompetent cells were transformed with 1000 ng pKDD001. Colonies were selected twice on LB + 50 mg/L kanamycin agar and twice on YT + 25% sucrose agar. The counterselected colonies underwent colony PCR with oKDD007 and oKDD008 to verify deletion of *znuA1* and a colony with the correct amplicon size of 217bp was selected to generate a strain stock.

[a]Cm^r, Amp^r, Km^r, resistance to chloramphenicol, ampicillin, and kanamycin, respectively.

[b]Numbers following "Tc1" indicate the location of the transposon insertion site (in bp) in the KT2440 genome.

Table S4. Synthetic DNA. Twist Biosciences was used for synthesis.

Name	Sequence (5'→3')	Description
**pACB127 insert**	TGTGCGCGTGCCTTTTACCGAGCTGCAAAGCCTGTTGCAGGCC<br>ATTTTCCAGCGCCATGGGTGCAGCGAGGCCGTGGCCCGGGTGC<br>TGGCCCACAACTGCGCCAGCGCCCAGCGCGATGGCGCCCATAG<br>CCATGGGGTGTTCCGCATGCCCGGTTATGTCTCGACCTTGGCC<br>AGCGGCTGGGTCGATGGCCAGGCCACGCCACAGGTCAGCGACG<br>TGGCCGCCGGCTATGTGCGCGTCGATGCTGCGGGCGGTTTTGC<br>CCAGCCGGCACTGGCGGCGGCCCGTGAGCTGTTGGTGGCGAAG<br>GCGCGCAGCGCAGGCATTGCCGTGCTGGCGATCCACAACTCGC<br>ACCATTTCGCTGCGTTGTGGCCGGATGTCGAGCCGTTCGCCGA<br>AGAGGGCCTGGTAGCCCTCAGCGTGGTCAACAGCATGACCTGC<br>GTGGTGCCGCATGGTGCACGCAAGCCGCTGTTCGGTACCAACC<br>CCATCGCTTTCGCTGCGCCTTGCGCCGAGCATGACCCGATCGT<br>TTTCGACATGGCCACCAGTGCCATGGCCCATGGCGATGTGCAG<br>ATTGCCGCACGTGCTGGCCAGCAATTGCCGGAGGGCATGGGGG<br>TAGATGCCGATGGCCAGCCGACTACCGACCCGAAGGCGATCCT<br>GGAAGGCGGGGCCTTGCTGCCATTTGGCGGGCACAAGGGCTCG<br>GCGTTGTCGATGATGGTCGAGCTGCTGGCGGCGGCGCTGACCG<br>GCGGTCATTTCTCCTGGGAGTTCGATTGGTCCGGGCATCCGGG<br>GGCGAAAACGCCATGGACCGGGCAGTTGATCATCGTCATCGAC<br>CCGGGCAAGGCCGAAGGCCAGCGGTTTGCCCAGCGCAGCCGCG<br>AGCTGGTGGAGCACATGCAGGCGGTGGGGCTGACGCGCATGCC<br>GGGCGAGCGGCGCTACCGTGAGCGCGAGGTGGCCGAGGAGGAG<br>GGGGTGGCGGTGACCGAGCAGGAGTTGAAAGGCCTGAAAGAGC<br>TGCTTGGCTGA**AGTCAAAAGCCTCCGACCGGAGGCTTTTGACT**<br>**GAGCTGTTGACAATTAATCATCGGCTCGTATAATGTGTGGAAT**<br>**TGTGAGCGGATAACAATTTCACACTTCCATCCATAGCCCTATA**<br>**TTCTCAAGGAGTATTTT**GTGTTCAAACATGTCGATGCCTATGC<br>CGGCGACCCGATCCTCTCGTTGATGGAAACCTTCAAGGCCGAC<br>CCGCGCGCCGACAAGGTCAACCTGAGTATTGGCCTGTACTACG<br>ATGAGGCCGGCGTGGTGCCGCAACTGGCGGCGGTGGATGCGGT<br>GGAAAAACGCATTGCCGGTCAGGACCACGAAGCCTCCCTGTAC<br>CTGCCAATGGAAGGCCTGGCCAGCTACCGCCAAGCCATCCAGG<br>CGCTGCTGTTCGGTGCTGATCACCCGGCCGTGACCGGCGGTCG<br>TGTGGCCACCGTACAGACCGTGGGTGGCTCCGGCGCCCTGAAA<br>GTCGGTGCCGATTTCCTCAAGCGCTACTTCCCGCAGTCCGAAG<br>TCTGGGTCAGCAACCCGACCTGGGACAACCACCGCGCCATCTT<br>CGAAGGTGCAGGCTTCAAGGTGCACACCTATCCGTACTTTGAC<br>CAGGCCACCCGTGGCGTGGACTTCGACGGCATGCTGGCCACCT<br>TGCAGAGCCTGCCGGCCAACAGCGTGGTACTGCTGCACCCGTG<br>CTGCCACAACCCTACCGGTGCCGATCTGCAGCAGCACCAGTGG<br>CAACAAGTGGTCGAAGTGGTCAAGGCACGCCAGCTGATCCCGT<br>TCCTCGACATCGCTTATCAAGGCTTCGCCGAAGGCCTGGTGGA<br>AGATGCCTACGCCATCCGCGAAATGGCCCGTGCCGGCGTGCCG<br>TGCCTGGTCAGCAACTCCTTCTCGAAAATCTTCTCGCTGTATG<br>GCGAGCGGGTGGGCGGCCTGTCGGTGGTGTGCGATGACGAGGC<br>CACTGCCCAGAGCGTGCTTGGCCAGCTGAAGGCCACCGTGCGC<br>CGCAACTACTCCAGCCCGCCCAACTTCGGCGCCCAGCTGGTTG<br>CCGGCGTACTGAGCGATGCTGGCCTGAATGCCCAGTGGGCCGA<br>AGAAGTCGAAGTGATGCGCAAGCGTATTCTCGACATGCGCCAG<br>GCGCTGGTCGATGCCCTGGCCGTGCTGC	Twist Biosciences synthesized DNA and cloned into the EcoRI/HindIII restriction sites of pK18sB. Gray text indicates up- and downstream genomic targeting regions for homologous recombination of the pACB127 plasmid. Underlined text indicates the native *amaC* CDS. **Bold blue text** indicates the TonB terminator sequence. **Bold red text** indicates the tac promoter sequence. **Bold green text** indicates the synthetic RBS designed for *amaC* (PP_3590) with the Salis lab RBS Library Calculator tool (3).

References

1. Cánovas D, Cases I, De Lorenzo V. 2003. Heavy metal tolerance and metal homeostasis in *Pseudomonas putida* as revealed by complete genome analysis. Environ Microbiol 5:1242-1256.

2. Bleem A, Kuatsjah E, Presley GN, Hinchen DJ, Zahn M, Garcia DC, Michener WE, König G, Tornesakis K, Allemann MN, *et al*. 2022. Discovery, characterization, and metabolic engineering of Rieske non-heme iron monooxygenases for guaiacol *O*-demethylation. Chem Catalysis 2:1989-2011.

3. Cetnar DP, Salis HM. 2021. Systematic quantification of sequence and structural determinants controlling mRNA stability in bacterial operons. ACS Synth Biol 10:318-332.

4. Wetmore KM, Price MN, Waters RJ, Lamson JS, He J, Hoover CA, Blow MJ, Bristow J, Butland G, Arkin AP. 2015. Rapid quantification of mutant fitness in diverse bacteria by sequencing randomly bar-coded transposons. MBio 6:e00306-15.
